# The impact of human health co-benefits on evaluations of global climate policy

**DOI:** 10.1038/s41467-019-09499-x

**Published:** 2019-05-07

**Authors:** Noah Scovronick, Mark Budolfson, Francis Dennig, Frank Errickson, Marc Fleurbaey, Wei Peng, Robert H. Socolow, Dean Spears, Fabian Wagner

**Affiliations:** 1 Department of Environmental Health, Rollins School of Public Health, Emory University, 1518 Clifton Rd. NE, Atlanta, GA 30322 USA; 2 Woodrow Wilson School of Public and International Affairs, Princeton University, Robertson Hall, Princeton, NJ 08544 USA; 3 Gund Institute for Environment and Department of Philosophy, University of Vermont, Burlington, VT 05310 USA; 4 Edmond J. Safra Center for Ethics, Harvard University, 124 Mount Auburn Street, Cambridge, MA 02138 USA; 5 Social Sciences (Economics), Yale-NUS College, Singapore, 138610 Singapore; 6 NUS fellow at the Center for Advanced Study in the Behavioral Sciences, Stanford, CA 94305 USA; 7 Energy and Resources Group, University of California Berkeley, 310 Barrows Hall, Berkeley, CA 94720 USA; 8 University Center for Human Values, Princeton University, 304 Louis Marx Hall, Princeton, NJ 08544 USA; 9 School of International Affairs and Department of Civil and Environmental Engineering, Pennsylvania State University, University Park, PA 16801 USA; 10 Department of Mechanical and Aerospace Engineering, Princeton University, Olden Street, Princeton, NJ 08544 USA; 11 Department of Economics, University of Texas at Austin, 2225 Speedway, Austin, TX 78712 USA; 12 Economics and Planning Unit, Indian Statistical Institute – Delhi Centre, 7, S.J.S Sansawal Marg, New Delhi, 110016 India; 13 IZA Institute of Labor Economics, Schaumburg-Lippe-Strasse 5-9, 53113 Bonn, Germany; 14 Institute for Futures Studies, Holländargatan 13, Stockholm, Sweden; 15 International Institute for Applied Systems Analysis, Laxenburg, 2361 Austria; 16 Andlinger Center for Energy and the Environment, Princeton University, 86 Olden Street, Princeton, NJ 08544 USA

**Keywords:** Environmental sciences, Environmental social sciences, Climate-change mitigation, Climate-change policy, Energy and society

## Abstract

The health co-benefits of CO_2_ mitigation can provide a strong incentive for climate policy through reductions in air pollutant emissions that occur when targeting shared sources. However, reducing air pollutant emissions may also have an important co-harm, as the aerosols they form produce net cooling overall. Nevertheless, aerosol impacts have not been fully incorporated into cost-benefit modeling that estimates how much the world should optimally mitigate. Here we find that when both co-benefits and co-harms are taken fully into account, optimal climate policy results in immediate net benefits globally, overturning previous findings from cost-benefit models that omit these effects. The global health benefits from climate policy could reach trillions of dollars annually, but will importantly depend on the air quality policies that nations adopt independently of climate change. Depending on how society values better health, economically optimal levels of mitigation may be consistent with a target of 2 °C or lower.

## Abstract (web summary)

Aerosol impacts have not been comprehensively considered in the cost-benefit integrated assessment models that are widely used to analyze climate policy. Here the authors account for these impacts and find that the health co-benefits from improved air quality outweigh the co-harms from increased near-term warming, and that optimal climate policy results in immediate net benefits globally.

## Introduction

Climate policies targeting CO_2_ may also reduce air pollutant emissions—and the aerosols they produce—as the two share emission sources. Prior studies on the topic have quantified the associated health co-benefits of pre-defined greenhouse gas reduction scenarios^1–6^, or estimated the economic impacts from reducing specific pollutants^7–9^, but these types of impacts have not been fully incorporated into cost-benefit modeling that estimates how much the world should optimally mitigate. We move this literature forward by developing a comprehensive cost-benefit integrated assessment model based on William Nordhaus’ Regionalized Integrated Climate Economy (RICE) model, where the new developments allow the model to weigh both the health co-benefits and the climate co-harms of aerosol co-reductions (co-harms exist because aerosols produce net cooling overall^10^); the latter in particular has been a largely neglected aspect of the co-benefits discussion. (We use the term co-harm to refer to the net climate harm of aerosol reductions, recognizing that reducing some species of emissions individually may produce different effects than the net of all species together; for example, although reducing black carbon produces a climate benefit, that effect may be outweighed by the climate harm of reductions in other species.).

These modeling developments, which account for the key air pollutant emissions and their individual properties, provide new capability to investigate fundamental policy questions that have not been answered by existing studies^11^. This includes determining: (1) the optimal climate policy across time and how it is affected by independent air quality control, (2) whether climate policy produces immediate net benefits, or if there are intergenerational tradeoffs, and (3) if specified climate targets are justifiable on cost-benefit grounds.

To answer these questions, we modify the RICE optimization model^12^ to include an empirically calibrated, regionally differentiated feedback mechanism whereby reducing CO_2_ also reduces regional air pollutant emissions from co-emitting sources. We then quantify and monetize the impact on both health and radiative forcing throughout the world and compute the resulting optimal climate policy. We thus modify the standard tradeoff between CO_2_ mitigation costs and climate damages with a more complete analysis that simultaneously weighs mitigation costs, climate damages from CO_2_, and the health and climate consequences of changes in air pollutant co-emissions. The resulting model estimates optimal climate policy after jointly considering all these factors. As a robustness check, we also modify the widely used FUND (Climate Framework for Uncertainty, Negotiation and Distribution) model^13^ to include the same mechanisms.

We find that when both co-benefits and co-harms are taken fully into account, optimal climate policy results in immediate net benefits globally, which overturns previous findings from cost-benefit models that omit these effects. The global health benefits from climate policy could reach trillions of dollars annually, but their magnitude will importantly depend on the air quality policies that nations adopt independently of climate change. Depending on how society values better health, we show that economically optimal levels of mitigation may be consistent with a target of 2 °C or lower.

## Results

### Main results

**Fig. 1:**
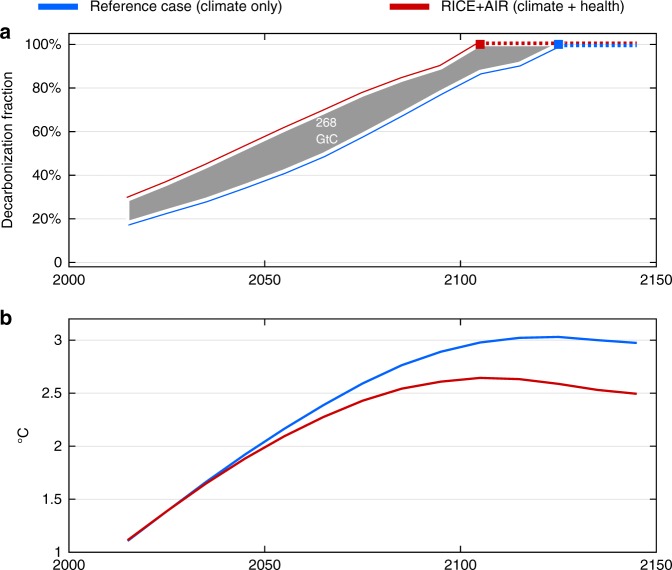
Optimal decarbonization and temperature. **a** Decarbonization over time for the reference case optimal policy that excludes health co-benefits (blue line) and in the full RICE + AIR optimal policy that includes health co-benefits (red line). **b** Estimated global temperature rise above preindustrial levels that would occur given the decarbonization in **a**. Decarbonization is relative to a business-as-usual scenario without any climate action, and 100% decarbonization signifies zero net carbon emissions

We summarize the results of the new model (hereafter referred to as RICE + AIR, for RICE + Aerosol Impacts and Responses) in terms of the optimal fraction of business-as-usual CO_2_ emissions that should be reduced over time. We refer to this quantitative reduction in CO_2_ as the optimal decarbonization fraction and express it as a percentage. (What we call the decarbonization fraction is often referred to as the control rate.). The blue line in Fig. 1a shows the optimal decarbonization pathway if the cost-benefit analysis only considers the climate impacts of CO_2_ and associated aerosol co-reductions. Health impacts are not included. In this reference case, the optimal decarbonization fraction is 24% of business-as-usual emissions in 2030, rising to 35% in 2050 and ultimately reaching full decarbonization by 2130. It is similar to the optimal trajectory of the standard RICE model, which features exogenous aerosol forcing and excludes health co-benefits (Supplementary Fig. 1).

In contrast to the blue line, which considers only the climate impacts of CO_2_ and aerosols, the red line also includes the health co-benefits of the aerosol reductions that result from decarbonization. This leads to increased optimal CO_2_ mitigation. The difference between the red line and the blue line demonstrates the importance of the health benefits; roughly 45–60% more decarbonization is optimal over the next five decades (and 10–40% thereafter) compared to the reference case that only considers climate consequences. Full mitigation also occurs earlier in time. The additional emission reductions that result from the inclusion of the health co-benefits cumulatively amounts to ~270 GtC (Fig. 1a). Importantly, all of these results account for the damages from lost cooling attributable to the aerosol co-reductions.

The additional decarbonization justified by the health gains leads to a peak temperature 0.4 °C lower than the reference case (Fig. 1b). The carbon price pathways associated with Fig. 1 can be found in Supplementary Fig. 2.

**Fig. 2:**
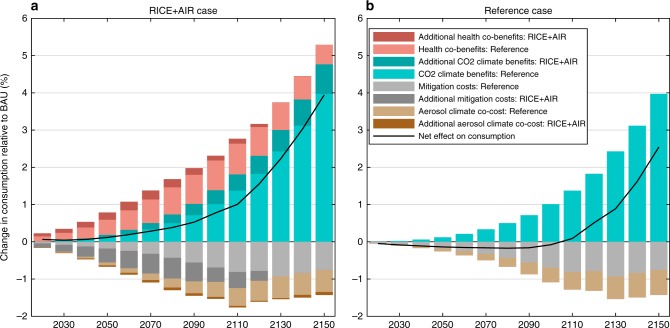
Costs and benefits of mitigation. Decomposition of the change in global consumption relative to the business-as-usual (BAU) scenario under **a** the full RICE + AIR optimal policy, and **b** the reference case optimal policy. Health co-benefits and benefits from avoided CO_2_ damages are positive, while mitigation costs and aerosol co-harms (climate damage from the co-reduction of cooling aerosols) are negative. The black solid line displays the global net effect. **a** shows that the net effect on global consumption is immediately positive when health co-benefits are taken into account, in contrast to the reference case (**b**), which is representative of standard cost-benefit models that do not include health co-benefits and thus imply that optimal climate policy has net costs for much of this century. If health co-benefits are added to the reference policy in **b**—by adding the light-red bars displayed in **a**—the global net effect becomes immediately positive, and if health co-benefits were removed from **a**, the net effect would be negative for most of this century

Figure 2a shows that the optimal climate policy has immediate and continual monetized global net benefits when accounting for health co-benefits. This overturns the findings from standard cost-benefit optimization models, which ignore health co-benefits and thus imply that optimal climate policy has net costs for much of this century (Fig. 2b). The result is consistent with prior co-benefits studies that have analyzed specific emission reduction scenarios and reported high benefits relative to mitigation costs^1,14^, but which generally do not also account for (monetized) climate-related impacts.

**Fig. 3:**
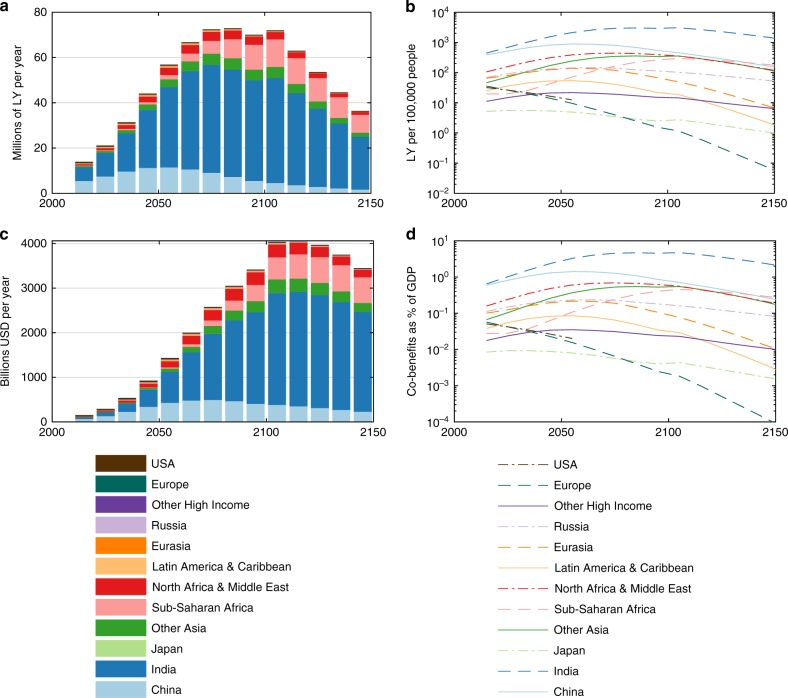
Health benefits of carbon mitigation. Life-years gained **a** overall and **b** per 100,000 population by region from the air quality improvements associated with the optimal decarbonization in RICE + AIR. **c**, **d** show the resulting monetized benefits in total, and as a percent of GDP, respectively. Note that if a region’s PM_2.5_ exposure (concentration) drops below 5.8 µg/m^3^, health benefits no longer accrue—this threshold assumption is common in other global air quality assessments^16^, and is tested in the sensitivity analyses

The distribution of health co-benefits by region for the full RICE + AIR optimum is displayed in Fig. 3a. In line with recent scenario-based studies^1,2,15^, many of the co-benefits accrue in India and China in early periods, attributable to their large populations and high capacity for mitigation-related reductions in PM_2.5_. China’s benefits decline by mid-century due to relatively rapid economic development and a stabilizing population—which both act to constrain emissions—whereas those in India persist and are the major driver of increased decarbonization relative to the reference case (see below for a sensitivity test which corroborates India’s importance). Towards the end of the century, sub-Saharan Africa replaces China as the second-largest beneficiary, as air pollution remains problematic due to lagging economic development accompanied by the world’s largest population. Other regions also stand to benefit, including less populous regions that show important benefits per capita and/or per Gross Domestic Product (GDP) (Fig. 3b).

In the results presented above, the averted premature mortality from aerosol co-reductions produces annual monetized benefits in the hundreds of billions of dollars over the next few decades, rising to several trillion annually at the end of the century (Fig. 3c). To derive these numbers, we multiply, for each region-time pair, the total life-years gained by 2 years of per capita consumption. This approach produces different life-year monetizations for each region, which leads to the slight change in composition of monetized benefits (compare Fig. 3a, c). However, this does not imply that we assign life-years in poorer regions less value in the objective function, because the optimization accounts for the diminishing marginal utility of consumption through a concave relationship between wealth and well-being, as described below and in the Methods section (Eq. (1)). Below we also show the sensitivity of our results to alternative valuations of health benefits.

### Independent air quality control

**Fig. 4:**
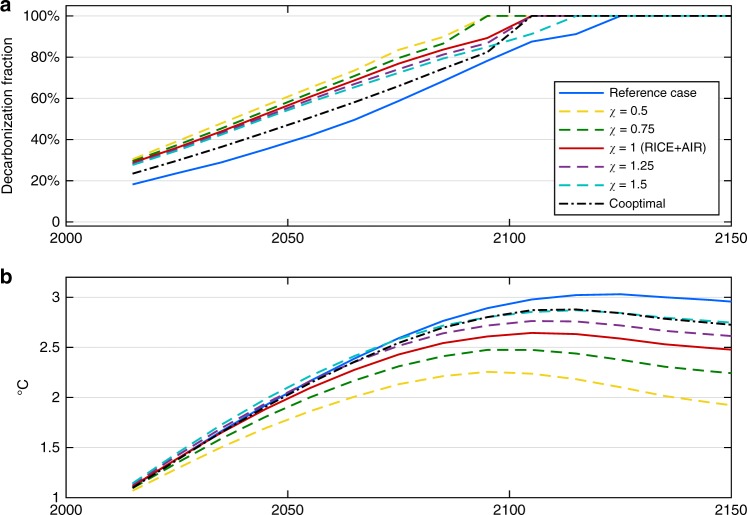
Impact of independent air quality control. **a** Optimal decarbonization and **b** associated global temperature rise given different levels of autonomous air quality control. The reference and RICE + AIR cases from Fig. 1, where *χ* = 1, are displayed as the blue and red solid lines, respectively

All results presented above assume the level of air quality control that occurs independently of climate policy will proceed approximately as projected in the coming decades, based on current and planned policies. We systematically test the importance of this assumption by implementing an environmental Kuznets-type approach where emission intensities decrease with increasing per capita income (described in detail in the Methods section, and in particular Eqs. (2)–(4) and associated discussion). Figure 4 reports results when we slow down and speed up this Kuznets-relevant income, allowing it to range between roughly 50% (*χ* = 0.5) and 150% (*χ* = 1.5) of the true (modeled) income; lower values imply less stringent air quality control and vice versa. In these model runs the true income is used in all other parts of the model.

The results demonstrate that although assumptions about air quality control do influence optimal mitigation levels, more decarbonization remains optimal compared to the reference case under all of these scenarios. However, the mechanisms driving the additional decarbonization vary. If the (pre-mitigation) air is dirtier than projected (*χ* < 1), extra mitigation occurs primarily to reap the health co-benefits. If stringent air quality control occurs in the future (*χ* > 1), more decarbonization is also optimal compared to the reference case. In part this is to capture remaining health co-benefits, but also for two other reasons. First, the potential damages associated with the substantial loss of the aerosol cooling effect requires additional CO_2_ reductions as a counterbalance. And second, when the air is cleaner, each marginal reduction in CO_2_ has a greater net benefit because it is not coupled to as much cooling aerosol.

Another way of understanding the role of independent air quality measures on optimal climate policy is by comparing not only the different RICE + AIR cases, as in Fig. 4, but also the difference between the RICE + AIR cases and their corresponding reference cases, as reported in Table 1. The results confirm that as autonomous emission controls get stronger (represented by higher *χ* values), the less health co-benefits matter for climate policy.

**Table 1:** Impact of independent air quality control

	2030	2030	2050	2050	Diff. in peak temp (°C)^a^
	Reference	RICE + AIR	Reference	RICE + AIR	
*χ* = 0.5	20%	39%	27%	57%	0.9
*χ* = 0.75	22%	37%	33%	54%	0.5
*χ* = 1 (main result)	24%	36%	35%	52%	0.4
*χ* = 1.25	24%	35%	36%	51%	0.4
*χ* = 1.5	25%	35%	37%	50%	0.3

Optimal decarbonization rates in RICE + AIR versus the corresponding reference case for different values of *χ*, which represent different levels of independent air quality control. *χ* = 1 represents the assumption underlying the main results, whereas higher (lower) values indicate more (less) stringent air quality control in the future. The difference in peak temperature is the difference between the highest global average temperature in the RICE + AIR optimum versus the reference case given the assumed value of *χ*

^a^Positive values indicate a lower peak temperature in RICE + AIR. Values are rounded

One of the scenarios displayed in Fig. 4 is the fully co-optimal case that selects the ideal combination of both air quality and climate policies by introducing an additional policy lever that can act directly on individual air pollutant emissions through end-of-pipe measures, rather than via CO_2_ (described in Supplementary Note 1). When this lever is added, large reductions in air pollutant emissions occur when and where associated abatement costs are low, the reductions lead to relatively large decreases in exposure, and/or they produce few climate damages. This first-best policy where climate and air pollution policies are co-optimized leads to rapid air quality improvements but still increased decarbonization relative to the reference case, albeit by a lower margin than in the standard *χ* = 1 case that underlies our main results. Note that we selected *χ* = 1 as the basis for the main result in order to explore the effect health co-benefits have when regions act consistently with their current and planned air pollution policies (which are suboptimal). As this section demonstrates, the magnitude of the co-benefit effect is importantly influenced by the assumed level of independent air quality control.

### Discounting

The RICE model has a discounted utilitarian objective, meaning that for optimal policy calculations, the objective of the model is to maximize the sum of discounted well-being (see Eq. (1) of the Methods and associated discussion). The discount rate for consumption is determined via the Ramsey rule, which adds the rate of pure time preference to the product of the elasticity of marginal utility and the economic growth rate. The rate of pure time preference is the rate at which the weight given to future well-being declines over time. The elasticity of marginal utility represents the lesser importance of each additional dollar to well-being as one gets richer. Unless otherwise noted, we assume an elasticity of marginal utility of 1.5, the default in RICE.

**Fig. 5:**
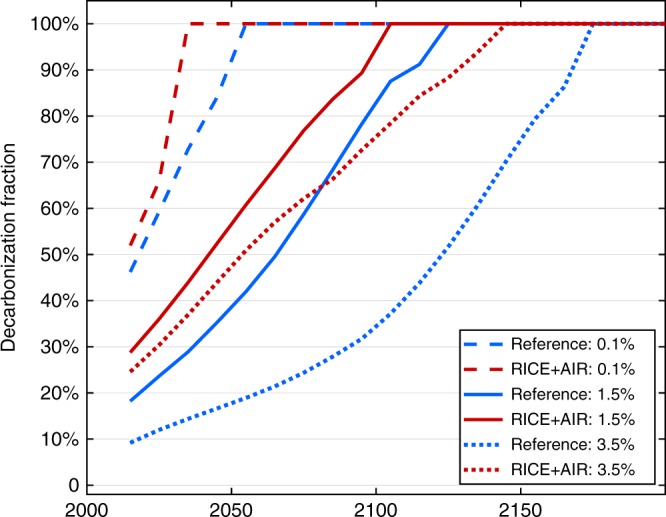
The influence of pure time preference. Optimal decarbonization with low (0.1%), medium (1.5%), and high (3.5%) rates of pure time preference for the reference case (that excludes health co-benefits) and in the full RICE + AIR case (that includes health co-benefits). All runs have a consumption elasticity of marginal utility of 1.5

In all the results presented so far we have used a rate of pure time preference of 1.5% per year, which is the default value RICE, but close to the upper end of the range highlighted by the IPCC (Intergovernmental Panel on Climate Change)^16,17^. This choice puts more weight on near-term impacts compared to those occurring further in the future. As a result, aerosol impacts, which occur more immediately than the climate impacts from CO_2_, have a relatively outsized importance. Nevertheless, when we implement a much lower rate that corresponds to a near-zero (0.1%) preference for the present over the future, optimal decarbonization remains substantially higher in RICE + AIR compared to the analogous reference case (Fig. 5). (Near-zero time preference is often used in the climate economics literature^17^, including in the Stern Review^18^).

Conversely, if we increase time preference to 3.5%, which implies a discount rate for consumption of 7%, the difference between RICE and RICE + AIR becomes even more stark. (To calculate the 7% discount rate via the Ramsey rule, we used the economic growth rate in the initial time period. We note that the exact discount rate may change over time due to the effect of climate damages on economic growth; the impact however, is negligible.). We chose the 7% value because it is the highest of the primary discount rates generally used in US federal cost-benefit analyses. (Although most experts consider lower rates to be more appropriate in an intergenerational context such as in the case of climate change-related analyses^16,17^, the current US administration has signaled a preference for including the higher 7% rate^19^.)

Such high time preference places so much emphasis on the near-term health benefits associated with reducing CO_2_ that optimal decarbonization is substantially higher than both the analogous reference case and the reference case with more moderate (1.5%) time preference. These results indicate that even after strongly discounting the future, a robust climate policy is still warranted. In Supplementary Fig. 3 we present further analyses showing results with a 3% discount rate, which is the lower value generally used for US federal cost-benefit analyses.

### Monetizing and valuing health benefits

The results above assume that one life-year gained equals 2 years of per capita consumption in dollar terms. Two years of per capita consumption corresponds to the approach to monetizing health impacts used in key studies that form the basis of the RICE climate damage function^12,20^, and is similar to survey-based life-year assessments^21^. However, this approach yields much lower monetary benefits than using the value of a statistical life (VSL), which is also widely adopted in the literature. If we assume that each adult death attributable to PM_2.5_ results in approximately 10 years of life lost^22^, we would use roughly 8–16 years of per capita consumption per life-year gained, instead of two; thus, in what follows we use 8–16 years of per capita consumption as one possible approximation of a VSL, in addition to a more direct VSL-based approach described below.

The sensitivity of our results to alternative life-year monetizations is reported in Table 2. The monetized global health benefits in our main results discussed above would be roughly 4–11 times higher if we used VSL-like monetizations in the optimization, and the associated optimal level of decarbonization would likely be consistent with keeping the maximum global temperature rise to 2 °C. Two degrees is a target specified in the Paris Agreement and widely considered as necessary to avoid dangerous climate change, but one that has generally not been warranted according to previous cost-benefit assessments (using similar discounting parameters) that omit health co-benefits^12^. Combining the low (0.1%) rate of time preference with a VSL-based approach may justify a target as low as 1.8 °C.

**Table 2:** Monetizing life-years/lives

	2030	2050	Peak temperature (°C)^a^
Reference (no co-benefits)	24%	35%	3.0
1 year of per capita income	30%	44%	2.9
2 years of per capita income (main result)	36%	52%	2.6
4 years of per capita income	46%	66%	2.3
8 years of per capita income	60%	85%	2.1
16 years of per capita income	78%	96%	1.9
VSL^b^	57%	85%	2.1
VSL with low discounting^c^	79%	100%	1.8

Optimal decarbonization rates in 2030 and 2050, as well as the maximum global temperature rise given different life-year monetizations for health co-benefits. (The last two rows use an alternative method that monetizes lives using the VSL.)

^a^Above preindustrial levels. Values are rounded

^b^Value of a statistical life. Equation: VSL = 9,000,000 × (GDPpc/54,000)^1.4^ as in Robinson et al.^23^

^c^In this run, time preference = 0.1%, compared to 1.5% in all other runs

Table 2 reports the sensitivity of the results to differences in how life-years are monetized in dollar terms. In RICE’s cost-benefit approach, an important second step occurs when the monetized health benefits are valued in well-being terms via the objective function (see Eq. (1) in the Methods section), which gives the model the aim of maximizing the (discounted) sum of global well-being through time, as is standard in optimal policy modeling. A key feature of the objective function is diminishing marginal utility of consumption, which captures the core concept that an additional dollar generates more well-being when given to a poorer person than to a richer person. The result is that while life-years are assigned a lower dollar amount in poorer countries in absolute terms, they are actually assigned greater value in well-being/utility terms. We discuss this issue in more detail in Supplementary Note 2 and show in Supplementary Table 1 that the strong effect of adding health co-benefits persists whether life years are valued more highly in poorer regions, less highly, or exactly the same as in wealthier regions; however, the magnitude of the effect changes.

### Additional sensitivities in RICE + AIR

Table 3 reports results for several other sensitivities, presenting the percent increase in optimal decarbonization rates in the RICE + AIR case compared to the corresponding reference case. The table is organized as follows, with variables in parentheses referring to the relevant parameter in the model equations reported in the Methods.

**Table 3:** Additional sensitivity analyses

	2030	2050	Diff. in peak temperature (°C)^a^
Main result	52%	49%	0.4
Test 1	7–49%	10–50%	0.1–0.4
Test 2	51%	48%	0.4
Test 3	33%	30%	0.2
Test 4	70%	65%	0.5
Test 5	52%	49%	0.4
Test 6	3%	13%	<0.1
Test 7	30%	29%	0.3
Test 8	82%	76%	0.7

Percent increase in optimal decarbonization rates in RICE + AIR versus the corresponding reference case given different modeling assumptions. The difference in peak temperature is the difference in the highest global average temperature

^a^The difference in peak temperature between the reference case and the RICE + AIR case within each test: positive values indicate a lower peak temperature in the RICE + AIR case. Values are rounded

First (Test 1), we explore different air pollutant co-reduction levels (*κ*), with the range representing the high and low values after applying alternative estimates from the other four Shared Socioeconomic Pathways (SSPs). Note that the SSPs are not ordered in terms of their co-reduction potentials, but instead reflect different possible futures across multiple socioeconomic dimensions. In the second test (Test 2), we substitute the TM5-FAst Scenario Screening Tool (TM5-FASST) source-receptor matrix (SRM) for the SRM based on simulations of the European Monitoring and Evaluation Program atmospheric chemistry and transport model. We confined this sensitivity to Asia because it was the only region analyzed as part of a recent project at the International Institute of Applied Systems Analysis. However, the four Asian regions account for ~85% of all life-years gained globally over the next century in the main analyses (Fig. 3), and therefore largely drive the findings. In Test 3 and Test 4 we assume that the relative risk for all-cause mortality (*β*) was the lower and upper bound, respectively, of the confidence interval in Forestiere et al.^24^ rather than the central estimate. Test 5 lowers the PM_2.5_ threshold below which there are no adverse health effects (*τ*) to 1 μg/m^3^ instead of 5.8 μg/m^3^. In Test 6 we assume there is no benefit from reducing air pollution at levels above 50 μg/m^3^. We include this counterfactual boundary case for two reasons. The first is to investigate the maximum possible concavity in risk functions at high levels of exposure; in reality however, recent empirical studies^25,26^ indicate that the marginal effect of air pollution remains positive at levels well above 50 μg/m^3^. The second reason is to provide additional emphasis on the importance of India and China, as this test effectively eliminates the impact of both of those nations (as well as the Middle East/North Africa region) over the next several decades and thus explores the model’s sensitivity to their exclusion. Test 7 assumes that climate damages are twice the standard values in RICE, while the final test (Test 8) uses the Finite Amplitude Impulse Response (FAIR) climate model (version 1.0)^27^ as an alternative to RICE’s native climate model (Supplementary Note 3 describes how we integrate the FAIR model).

### Exploring model uncertainty by linking AIR to the FUND model

**Fig. 6:**
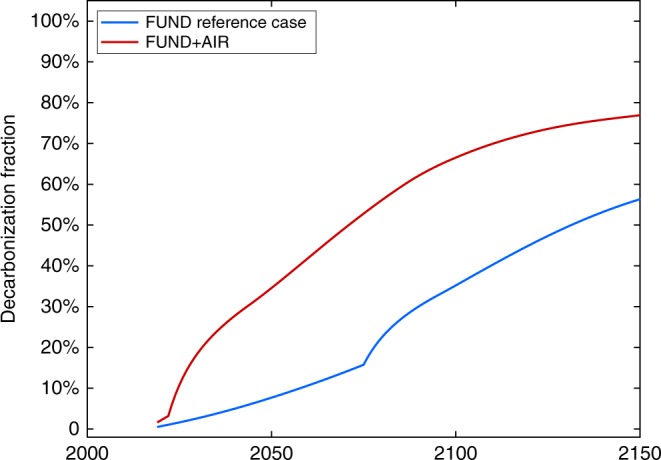
FUND + AIR results. Optimal decarbonization rates over time for the FUND reference case (that excludes health benefits) and the FUND + AIR case (that includes health benefits)

Like RICE/DICE (the Dynamic Integrated Climate Economy model (DICE) is RICE’s global counterpart), the FUND model is another one of the three leading climate economy models used by the US Interagency Working Group to estimate the social cost of carbon^28^. FUND has different world regions, a different economic framework, a different climate model, and a different specification of damages when compared to RICE, and thus provides an important opportunity to explore model uncertainty. Comparing Fig. 1 with Fig. 6 demonstrates that results for FUND + AIR and RICE + AIR are qualitatively similar, despite the well-known differences in the structure and policy recommendations of the two models^28,29^; adding aerosol impacts leads to dramatically increased optimal levels of decarbonization. (Supplementary Note 4 describes how we link FUND to AIR.)

## Discussion

We have developed a new modeling framework for analyzing the costs and benefits of the co-reductions in air pollutant emissions that result from CO_2_ policy. We find that these impacts have a critical role in determining optimal decarbonization rates, as the potential health co-benefits that result from improved air quality are large, occur quickly enough to be economically important, outweigh the near-term co-harms from lost cooling, and are concentrated in developing regions. This remains true even with high discount rates and relatively conservative valuations of improved health. However, as the size of the health co-benefits will be partially determined by future air quality policies, decision makers should jointly plan both types of interventions. Depending on how society values health, it may be economically optimal to limit temperature rise to 2 °C or lower, thus corroborating the climate targets from the Paris Agreement. Overall, optimal mitigation results in immediate net benefits globally.

Our findings should be interpreted in light of several factors. First, our sensitivity analyses identified key variables that meaningfully affect the optimal level of decarbonization in RICE + AIR. These include the shape and magnitude of the exposure-response functions that relate PM_2.5_ exposure to mortality, the assumed relationship between CO_2_ emissions and air pollutant emissions, the discount rate, and the valuation of the health benefits. The first two factors are largely amenable to empirical inquiry, whereas the latter two depend partially on ethical judgements; all have a strong bearing on how much society should mitigate, and when.

Second, we follow standard convention in assuming a uniform global carbon price in our optimal policy calculations. Economists prefer this assumption because a uniform price minimizes the cost to the global economy of any particular level of emissions reductions, and is thus a necessary part of the first-best approach to climate policy. However, another necessary part of first-best policy is a non-climate equity policy involving redistribution to address the large economic inequalities that exist throughout the world; in the absence of such an equity-focused policy, a uniform global carbon price would ignore equity considerations, and arguably impose an unjustifiably heavy burden on developing countries. An important feature of our results relevant to this issue is that health co-benefits provide the most incentive for additional decarbonization in lower- and middle-income regions. An interesting extension of our research would explore how co-benefits affect mitigation policies in a range of different burden-sharing regimes and in second-best climate policy calculations^30,31^.

Third, we follow common practice in the co-benefits literature in assuming that there is a background appetite for air pollution policy that is independent of climate policy, such that the same background levels of investment will be made in air quality regardless of CO_2_ reductions (and so policies will ratchet up insofar as greater CO_2_ reductions make the antecedent level of air quality mitigation less expensive); this is the basic assumption behind the co-reductions we estimate in our main results. As reported above in Fig. 4, we also co-optimize air quality and climate policies, which is another scenario (with different properties) in which there would be co-benefits from CO_2_ reductions. A limitation is that other scenarios are also possible, such as CO_2_ reductions in a context involving a fixed cap and trade regime for air pollutants where the cap remains fixed at the same level in conjunction with new CO_2_ reductions; in this scenario, it is possible that there would not be many co-benefits from CO_2_ reductions^32^.

Fourth, we were not able to fully assess uncertainties around key aerosol processes. For example, the aerosol indirect effect may be the single largest source of uncertainty in radiative forcing assessments^10,33^. New evidence continues to accrue about key factors that contribute to the formation of new particles from precursor gases, and there are likely to be complex feedback mechanisms that occur in response to future temperature changes^34,35^. Some of these processes are not yet adequately represented in even the most sophisticated Earth system models, let alone reduced-form versions. A related concern is that we have used separate models to estimate the health effects (TM5-FASST) and climate effects (Model for the Assessment of Greenhouse gas Induced Climate Change 6 (MAGICC6)) of air pollutant emissions, a decoupling that may also introduce uncertainty. Nevertheless, MAGICC and TM5 have distinct strengths that we harness accordingly; MAGICC takes account of the full aerosol load of the whole atmosphere, top to bottom, and at the level of hemispheres, while TM5 tells us about the concentration at ground level, where people live and breathe, in principle at much higher resolution. Both models work with the same global emission inventories.

Fifth, we have only explored the effect of co-reducing air pollutant emissions on PM_2.5_-related mortality. Other co-benefits may occur, which would likely push further in the direction of increased decarbonization; these include morbidity impacts from PM_2.5_—which are generally minor compared to those from mortality—as well as other more indirect impacts such as potential increases in crop yields, improvements in visibility, and health impacts from changing exposure to tropospheric ozone^2,9,35,36^. In addition, cost-benefit models also miss some other impacts of reduced climate change, including the effects of methane on ozone, the effects of ocean acidification, and others^37^.

Due to the uncertainties inherent in our modeling framework, we expect the accuracy of quantitative estimates of the co-harms and co-benefits of optimal climate policy to sharpen over time, particularly as our understanding of atmospheric science progresses. Nevertheless, the novel modeling approach described here offers important new insights into how much we should mitigate and over what time period, and the sensitivity tests above indicate that we should not expect the qualitative story told by our results to change in light of improved empirical estimates. Our methods also enable investigation of other key questions beyond the scope of this study, including how the inclusion of health co-benefits influences optimal climate policy under different burden-sharing regimes and different worldviews about how much to prioritize the poor, future people, and citizens of other countries.

## Methods

### The RICE model

The RICE model was first developed in 1996 to analyze the tradeoffs between investing in climate mitigation, which incurs a cost relatively soon, and climate damages, which incur costs in the more distant future^38,39^. RICE is the regionalized counterpart to the DICE model, which is one of three leading cost-benefit climate economy models used by researchers and governments for regulatory analysis, including to estimate the social cost of carbon^28^. Here we describe the key aspects of the standard RICE2010 model; for a more extensive description of this open-access model, see ref. ^12^. (Also see ref. ^39^ for a more extensively documented, but earlier version of RICE.)

Briefly, RICE is a regionalized global optimization model that includes an economic component and a geophysical component that are linked. RICE divides the world into 12 regions, some of which are single countries, while others are groups of countries. Each region has a distinct endowment of economic inputs, including capital, labor, and technology, which together produce that region’s gross output via a Cobb–Douglas production function. Pre-mitigation carbon emissions are a function of gross output and an exogenously determined, region-specific carbon intensity pathway. These carbon emissions can be reduced (mitigated) at a cost to gross output through control policies, set to equalize the marginal abatement cost in all regions. Local mitigation cost is borne by each region, and there are no inter-regional transfers. Any remaining (post-mitigation) carbon emissions are incorporated into the climate module where they influence global temperature and, ultimately, the future economy through climate-related damages. Future climate change affects regions differently, with poorer regions generally more vulnerable to climate damages. Damage estimates increase quadratically with a change in the global surface temperature and, like mitigation costs, are incurred directly as the loss of a proportion of gross output. Gross output minus the loss of mitigation costs and climate damages is what we refer to hereafter as GDP.

The model’s optimization balances mitigation costs, which lower consumption at the time of mitigation, against climate damages which lower consumption in the future. (Regional consumption is the fraction of GDP that is not saved; mitigation cost and climate damage affect consumption only via their effect on GDP). The optimal tradeoff maximizes the sum of discounted well-being, *W*, which is a concave function of consumption as follows:

1 $$W(c_{it}) = \mathop {\sum}\limits_{it} {\frac{{L_{it}}}{{(1 + \rho )^t}}} \frac{{c_{it}^{(1 - \eta )}}}{{1 - \eta }},$$

where *L* is population, *c* per capita consumption, *ρ* the rate of pure time preference, and *η* the consumption elasticity of marginal utility (inequality aversion). The subscripts *i* and *t* are the region and time indices, respectively. The model is solved by maximizing this global objective function. As a result, any factor affecting consumption, such as health impacts or climate damages, can be included in the model’s optimization framework. Unless otherwise specified, we maintain RICE’s default parameter values for time preference and inequality aversion of 1.5% and 1.5, respectively.

For this study, all simulations were run using the Mimi model development package in the Julia programming language. This Mimi/Julia version of RICE is fully faithful to the standard Excel version, but has more flexibility^40^. We make four changes to this standard version of RICE, in addition to those directly related to the AIR module, which are described below. First, we update the population projections to those of the UN2017 medium variant^41^. This is a newer source of projections and also enables us to use internally consistent projections of deaths and life-years, as described further below. The impact of changing population has been comprehensively explored elsewhere^42–44^. Second, we update the exogenous radiative forcing terms to the values used in RCP6.0^45^, which is in line with the latest versions of DICE^46,47^, which is the global (single-region) variant of RICE. These estimates are newer than those found in RICE2010 and are available in disaggregated form, thus allowing us to remove and endogenize the individual aerosol term for use in the AIR module, while maintaining the other non-CO_2_ forcings (also described in more detail below). Third, we allow CO_2_ concentrations and the global temperature to be endogenous in the second time-step (2015–2025)—it is fixed in standard RICE2010—as the now-endogenized aerosols will produce effects immediately after mitigation. And fourth, we use a modified objective function that avoids Negishi weights, which distort time preferences^48^ and the inter-regional tradeoff in ways that are opaque and difficult to justify descriptively and normatively^49^. We have explained this latter change in more detail in a previous publication^50^ and also in a sensitivity presented in Supplementary Table 2.

In the standard RICE2010 model, anthropogenic CO_2_ is the only endogenous climate forcer. All other sources of radiative forcing, including land-use change, non-CO_2_ gases, and aerosols are represented through a single exogenous forcing term that aggregates the individual trajectories of each source. This simplifying assumption is problematic when it comes to aerosols. Mitigation actions affecting CO_2_ have the potential to strongly influence emissions of the air pollutants that produce aerosols, as the two types of emissions share many sources^4,5^. Therefore, if carbon emissions are reduced, aerosols will tend to decrease simultaneously. A change in aerosols implies a change in radiative forcing as well as a change in ambient particulate air pollution^5^. Climate change and air pollution both affect well-being, and capturing the impact of these pathways was the motivation for developing the AIR module, which we now explain.

### Overview of the AIR module

In this section, we provide a general overview of how we developed the AIR module, with a technical description—including all equations—in the sections that follow.

Broadly speaking, our approach consists of five steps. First, we estimate the baseline (before carbon mitigation) emissions of five air pollutant species (primary PM_2.5_, oxides of nitrogen, sulfur dioxide, organic carbon, and black carbon). Emissions are estimated for each region-time pair with income-dependent emission intensity projections (emissions per unit GDP) based on the Greenhouse gas and Air pollution Interactions and Synergies (GAINS) model and specifically the ECLIPSE emission scenarios^51^. Our central case assumes air pollutant emissions in the coming decades follow the ECLIPSEV5a baseline scenario, which includes current and planned air quality legislation but no climate policy. In sensitivity analyses we alter this assumption, allowing for faster or slower independent air quality cleanup, including a case where we simultaneously co-optimize both air quality and climate policy. This co-optimization introduces policy levers for end-of-pipe technologies that act on individual air pollutants. These levers require associated cost curves that are also drawn from the GAINS model.

In the second step, we determine the change in air pollutant emissions that would result from a change in CO_2_ emissions using information from the SSP project^52^. This provides an estimate of co-reductions based on empirically realistic projections about the regionally differentiated interaction between future climate and air pollution policies, and is consistent with theoretical results from economics that show that co-benefit effects could be different in scenarios with different properties^32^. Emission information in the SSPs is estimated from bottom-up integrated assessment modeling that includes regionally differentiated, spatially explicit representations of energy production and structure and, like in RICE, assumes that CO_2_ policy occurs through a single global carbon price.

Third, we link changes in air pollutant emissions to changes in estimated average human exposure to PM_2.5_ by applying the source-receptor matrix (SRM) from the TM5-FASST air quality model^53^. The TM5-FASST SRM was computed from simulations of the full TM5 chemical transport model for 56 source regions, which were aggregated to approximate the RICE regions^53^. Once exposure is estimated, it is possible to calculate the number of life-years gained attributable to (reduced) air pollution by combining an exposure--response function with projections of future mortality and life expectancy^54^, which we took from the UN World Population Prospects. We applied a (log)linear exposure-response function for mortality from all causes in adults based on a meta-analysis published in a recent World Health Organization report^24^. We selected this approach for consistency with the UN projections—which only estimate mortality from all causes—and because recent epidemiological analyses indicate that the strong effects of air pollution occur at exposure (concentration) levels up to and including those most relevant to our study and that they likely affect a wide range of outcomes^25,26^.

Fourth, we allow the change in air pollutant emissions to influence the global temperature using aerosol forcing coefficients derived from the MAGICC climate model^55^. The coefficients incorporate both the direct and indirect effects represented in MAGICC, with the latter including those related to albedo and cloud responses. Aerosol forcing is added to the forcing from the other greenhouse gases in RICE’s climate module to produce estimates of future climate change.

And fifth, for each region we monetize and then value the aerosol impacts. The average per capita health benefits are added to per capita consumption, whereas the climate effects—monetized using RICE’s standard climate damage function—subtract from consumption^12^. Consumption is transformed into well-being by a concave function in the optimization via RICE’s objective/social welfare function (Eq. (1)). Once the impacts have been valued in this way, they then enter the optimization.

**Fig. 7:**
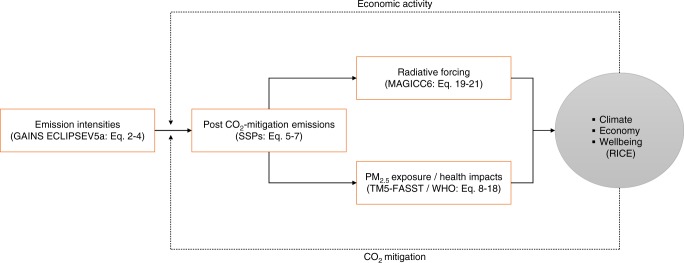
Diagram of the AIR module. Flow chart illustrating how the AIR module (rectangles) links with the RICE model (gray circle) to estimate emissions of air pollutants and their impacts. The model/method underlying each step in AIR is shown within parentheses, along with the relevant equations

Figure 7 illustrates the different model components and their linkages. We now present a technical description of each of these steps.

### Estimating baseline emissions

Air pollutant emissions from natural sources and open burning remain exogenous in our framework, following the trajectory based on RCP6.0^45^. All other anthropogenic air pollutant emissions are endogenous, as follows.

The level of baseline (pre-mitigation) anthropogenic emissions of each aerosol precursor, *E*^0^, is a function of an emission intensity factor (emissions per unit GDP), *e*, and the GDP, *Y*:

2 $$E_{itp}^0(Y_{it},L_{it},e_{itp}(Y_{it}/L_{it})) = e_{itp}(Y_{it}/L_{it})\cdot Y_{it}.$$

The emission intensity is region (*i*), time (*t*), and pollutant (*p*) specific and characteristically depends on the per capita income level of the region, defined above as the GDP, *Y*, divided by the population, *L*. GDP is endogenously determined in RICE while population is exogenous, taking the medium variant estimate of the 2017 version of the UN Population Prospects through 2100^41^, and remaining constant thereafter.

We estimated emission intensities (and emissions) for five air pollutants: sulfur dioxide (SO_2_), primary fine particulate matter (PM_2.5_), oxides of nitrogen (NO_*x*_), organic carbon (OC), and black carbon (BC). Emission intensities up to the year 2050 were derived from region-specific projections used in the GAINS model and specifically the ECLIPSEV5a scenario that reflects existing national and regional air pollution policies, but excludes decarbonization from climate mitigation^51^.

We fit the following functional form to the scenario data to extrapolate beyond 2050:

3 $$\begin{array}{c}e_{itp}(Y_{it}/L_{it},\chi ) = \varphi _{1,ip}\cdot \left[ {\exp \left( { - \Omega _{1,ip}\cdot I_{it}(Y_{it}/L_{it},\chi )} \right)} \right.\\ \left. { + \varphi _{2,ip}\cdot \exp \left( { - \Omega _{2,ip}\cdot I_{it}(Y_{it}/L_{it},\chi )} \right)} \right],\end{array}$$

where the Ωs and *φ*s are fitting parameters and *χ* is the Kuznets-relevant income, as described below. Resulting emission intensities are displayed graphically in Supplementary Fig. 4.

This functional form implies that emission intensities decrease with rising per capita income *I*, as observed in the projected emission scenario. This can be interpreted as a particular version of the environmental Kuznets curve, estimated with the ECLIPSE data. We write our Kuznets-relevant income *I*(*χ*) as:

4 $$I_{it}(Y_{it}/L_{it},\chi ) = \frac{{Y_{i(t = 2005)}}}{{L_{i(t = 2005)}}} + \chi \cdot \left( {\frac{{Y_{it}}}{{L_{it}}} - \frac{{Y_{i(t = 2005)}}}{{L_{i(t = 2005)}}}} \right).$$

For *χ* = 1, the Kuznets-relevant income equals the true (modeled) per capita income in the region-time pair, producing our default best-guess emission intensity factors. Changing *I*(*χ*) allows us to explore assumptions of more or less stringent autonomous air quality policies: we can speed up (*χ* > 1) or slow down (*χ* < 1) the decrease in emission intensities over time accordingly.

Supplementary Figure 5 shows the baseline (pre-mitigation) level of emissions by region and time under the default of *χ* = 1, where the Kuznets-relevant income equals the true (modeled) income.

### Relationship of CO_2_ mitigation to air pollutant emissions

The above method projects the level of air pollutant emissions assuming no CO_2_ mitigation. Reducing emissions of CO_2_ will typically also reduce the emissions of air pollutants, as they often stem from the same sources. In our framework, the percentage reduction in CO_2_ relative to the business-as-usual (without mitigation) scenario is called the decarbonization fraction (control rate), *μ*_*it*_, and it is associated through parameter *κ*_*ip*_ with a reduction in pollutant *p* relative to its baseline (pre-mitigation) level:

5 $$\frac{{\Delta E_{itp}(\mu _{it})}}{{E_{itp}^0}} = \kappa _{itp}\frac{{\Delta E_{it,{\mathrm{CO}}_{\mathrm{2}}}(\mu _{it})}}{{E_{it,{\mathrm{CO}}_{\mathrm{2}}}^0}} = \kappa _{ip}\cdot \mu _{it}$$

or,

6 $$\Delta E_{itp}(\mu _{it},Y_{it},L_{it}) = \kappa _{ip}\cdot \mu _{it}\cdot E_{itp}^0 = \kappa _{ip}\cdot \mu _{it}\cdot e_{itp}(Y_{it}/L_{it})\cdot Y_{it}.$$

The parameter *κ*_*ip*_ describes the effectiveness of CO_2_ mitigation in co-reducing emissions of pollutant *p*. The *κ*_*ip*_ parameter was estimated from the SSPs, which are a set of five storylines designed to analyze tradeoffs between climate change and socioeconomic factors^52^. Each of the five SSPs contains multiple sub-scenarios that differ only by their level of decarbonization; all socioeconomic factors remain constant. Therefore, each pairwise comparison of these sub-scenarios includes an implicit estimate of *κ*_*itp*_ at each time period for each region and pollutant. The emission information in the SSPs is estimated from bottom-up integrated assessment modeling that includes regionally differentiated, spatially explicit representations of energy production and structure, and like RICE, assumes that CO_2_ policy occurs through a single global carbon price.

We fit a simple linear regression line through the multiple estimates of *κ* for each SSP, constrained to begin at the origin, and take the slope of that line as our estimate (Supplementary Fig. 6). We derive five estimates of *κ* (one for each SSP) for each region-pollutant pair, using the middle-of-the-road SSP2 as our standard case, with the alternative SSPs tested in sensitivity analyses. The total post-mitigation level of emissions is therefore:

7 $$E_{itp}(\mu _{it}) = E_{itp}^0 - \Delta E_{itp} = E_{itp}^0\cdot (1 - \kappa _{ip}\cdot \mu _{it}).$$

We acknowledge that there are cases where the data derived from the SSP database appear to exhibit a non-linear rather than linear relationship between CO_2_ reduction and air pollutant reduction (Supplementary Fig. 6). Our goal was to find a relationship that on average, and in particular at full mitigation, provides a reasonable approximation to the implied air pollution reduction. Having said this, we are aware that our assumption of linearity in the relation may affect our overall results: a higher co-reduction at low CO_2_ mitigation offers incentives for further reduction than in the linear case, and vice versa. Thus, the shape of the mitigation profile could be affected, though the effect is likely to be small if, as we assume, the carbon prices in the RICE regions are coupled to each other.

### From air pollutant emissions to health impacts

The health co-benefits in RICE + AIR are calculated directly from the change in ambient population-weighted concentrations of PM_2.5_ attributable to CO_2_ mitigation. In the next paragraph, we describe how we estimate this change in concentration. Further below we describe how we keep track of the absolute level of pre- and post-mitigation PM_2.5_ concentrations, which are used in the health impact calculations only to ensure that no health benefits accrue at exposures below given threshold values.

The change in ambient concentrations of PM_2.5_ attributable to CO_2_ mitigation is a function of the change in aerosol precursor emissions—in this case primary PM_2.5_, NO_*x*_, and SO_2_—as well as other factors such as meteorological conditions. To estimate this relationship, we extracted the SRM from the freely available TM5-FASST global atmospheric SRM^53^. For each pollutant, the SRM provides an estimate of the change in population-weighted PM_2.5_ concentrations (hereafter referred to as exposure) given a unit change in emissions. The SRM from TM5-FASST was computed for 56 source regions from simulations with the full TM5 chemical transport model^53^. Using an SRM is a practical alternative to running full atmospheric chemistry transport model simulations, which is infeasible in our optimization context. The TM5-FASST model is described in detail elsewhere, and has been used similarly in other projects^3,53^.

The TM5-FASST interface allows the 56 source regions to be aggregated into larger regions that approximate the RICE regions. Due to the size of the RICE regions, we assume that the change in exposure to PM_2.5_, Δ*C*, in region *i* depends only on the change in emissions within that same region (*i*') and that the change is estimated via the SRM (SR) that encodes atmospheric transport. We also assume that the SRM does not change over time and that changes in the three precursor pollutants are additive:

8 $$\begin{array}{c}\Delta C_{it}(\mu _{it},Y_{it},L_{it}) = \mathop {\sum}\limits_{i{\prime}p} {{\mathrm{SR}}_{ii{\prime}p}} \cdot \Delta E_{i{\prime}tp}(\mu _{i{\prime}t},Y_{i{\prime}t},L_{i{\prime}t})\\ = \mathop {\sum}\limits_p {{\mathrm{SR}}_{iip}} \cdot \Delta E_{itp}(\mu _{it},Y_{it},L_{it}).\end{array}$$

As mentioned above, we keep track of the absolute level of exposure, a variable that is used only to ensure that no health benefits accrue at exposures below a given threshold level (described further below). The absolute exposure levels in the pre-mitigation case (where *μ* = 0) are calculated by translating the change in emissions relative to 2005 into a change in exposure using the SRM, and then subtracting it from the 2005 exposure

9 $$C_{it}^0(\mu _{it} = 0) = \max \left( {C_{i,t = 2005} - \mathop {\sum}\limits_{i{\prime}p} {{\mathrm{SR}}_{ii{\prime}p}} \cdot (E_{i{\prime},t = 2005,p} - E_{i{\prime}tp}^0),0} \right).$$

Here the max function ensures PM_2.5_ concentrations do not drop below zero. Emissions and exposure in 2005 were taken from the EDGAR (Emission Database for Global Atmospheric Research) emission database^56^ and Brauer et al.^57^, respectively, and then aggregated into the RICE regions.

Mitigation of CO_2_ (*μ* > 0) reduces exposure further:

10 $$C_{it}(\mu _{it}) = \max \left( {C_{i,t = 2005} - \mathop {\sum}\limits_{i{\prime}p} {{\mathrm{SR}}_{ii{\prime}p}} \cdot (E_{i{\prime},t = 2005,p} - (E_{i{\prime}tp}^0 - \Delta E_{i{\prime}tp})),0} \right)$$

11 $$= \max \left( {C_{i,t = 2005} - \mathop {\sum}\limits_{i{\prime}p} {{\mathrm{SR}}_{ii{\prime}p}} \cdot (E_{i{\prime},t = 2005,p} - E_{i{\prime}tp}(\mu _{it})),0} \right)$$

We define the health co-benefit as the avoided premature mortality resulting from reductions in PM_2.5_ exposure attributable to CO_2_ mitigation (Δ*C* from Eq. (8)). This benefit can be quantified through the attributable fraction (AF) (^54^):

12 $${\mathrm{AF}}_{it} = \frac{{{\mathrm{RR}}_{it} - 1}}{{{\mathrm{RR}}_{it}}},$$

where the relative risk, RR, for each region and each time period is a function of the change in exposure and a health impact function (*β*) that links a unit change in exposure to a change in the risk of adult (≥30) mortality from all causes:

13 $${\mathrm{RR}}_{it} = \exp (\beta \cdot \Delta C_{it}(\mu _{it},Y_{it},L_{it})).$$

We assume a log-linear relationship between PM_2.5_ exposure and all-cause mortality with a relative risk of 1.066 (95% confidence interval (CI) = 1.040, 1.093) for each 10 μg/m^3^ change in exposure, based on a meta-analysis published by the World Health Organization^24^. However, we note that many recent assessments of ambient air pollution have used the cause-specific integrated exposure-response (IER) functions to estimate mortality impacts^58^. Here we focus on all-cause mortality for two reasons. First, we have recently shown that population size/growth strongly affects estimates of optimal climate policy, including for reasons unrelated to human health^42,43^. Therefore, we use the most recent long-term (to 2100) population projections provided by the UN Population Division, which does not publish corresponding estimates of cause-specific mortality. Second, important recent studies indicate that the IER functions may underestimate excess mortality^25,26^, and suggest that mortality risks at the exposure levels seen in our regional analyses may fall within the 95% CI of the WHO estimate presented above and tested in sensitivity analyses^25^.

In all analyses, we assume that components of PM_2.5_ are equally toxic^24^, that health benefits accrue in the same 10-year time-steps as the improvement in air quality, and that population and mortality remains constant after 2100. We also confine the health impacts to mortality from PM_2.5_ exposure, though note that there is also concern about the health effects of exposure to smaller particles^59^.

Multiplying the AF by the total number of life-years lost from all causes, Θ, yields the life years gained from the reduction in air pollution:

14 $${\mathrm{LY}}_{it}(\mu _{it},Y_{it},L_{it}) = {\mathrm{AF}}_{it}\cdot \Theta _{it},$$

15 $$= \frac{{{\mathrm{RR}}_{it} - 1}}{{{\mathrm{RR}}_{it}}}\cdot \Theta _{it},$$

16 $$= (1 - \exp ( - \beta \cdot \Delta C_{it}(\mu _{it},Y_{it},L_{it})))\cdot \Theta _{it}.$$

Since *β* ⋅ Δ*C*_*it*_(*μ*_*it*_, *Y*_*it*_, *L*_*it*_) ≪ 1, we can write as an approximation:

17 $${\mathrm{LY}}_{it}(\mu _{it},Y_{it},L_{it}) = \beta \cdot \Delta C_{it}(\mu _{it},Y_{it},L_{it})\cdot \Theta _{it}.$$

Theta (Θ) values are estimated from the UN data by multiplying the total deaths by the remaining life expectancy at the age of death. As UN life expectancy data is by exact age, reported at 5-year intervals, whereas mortality data is for 5-year age groups, remaining life expectancy for each 5-year age group was taken as the average of the group’s bounding ages. For example, remaining life expectancy for all (averted) deaths in the 30–34 age group would be the average of the remaining life expectancy for a 30 year old and a 35 year old.

Health benefits in a given region can accrue until absolute exposure to PM_2.5_ converges to some minimum level, representing either a point below which no additional health impacts occur (a threshold) or a theoretical minimum level where residual PM_2.5_ consists only of natural sources. We followed recent global studies (e.g., ref. ^60^) and chose a lower threshold/theoretical minimum of 5.8 μg/m^3^. However, we acknowledge that there is a growing consensus that there may not be a safe level of PM_2.5_ below which no adverse health effects occur^59,61^ and therefore ran sensitivities down to 1 μg/m^3^. If a reduction in a given time period brings a region’s exposure below the threshold, the health co-benefit is calculated from the increment between the unmitigated level and the threshold:

18 $$\begin{array}{c}{\mathrm{LY}}_{it}^ \ast (\mu _{it},Y_{it},L_{it}) = \beta \cdot \Theta _i\cdot\max \left[ {\min \left( {\Delta C_{it}(\mu _{it},Y_{it},L_{it}),} \right.} \right.\\ \left. {\left. {(C_{it}^0(\mu _{it},Y_{it},L_{it}) - \tau )} \right),0} \right],\end{array}$$

where *τ* is the value of the threshold level.

### Radiative forcing and temperature effects from aerosols

Some air pollutants are climate forcers: BC is a warming agent while SO_2_, NO_*x*_, and OC act to cool the atmosphere^10^. The net global forcing in each time period attributable to aerosols is taken as the sum of the individual contributions:

19 $${\mathrm{RF}}_t^{{\mathrm{aer}}} = \mathop {\sum}\limits_p {\mathop {\sum}\limits_i {r_{ipt}} } \cdot {\mathrm{E}}_{itp}(\mu _{it},Y_{it},L_{it}),$$

where *r*_*ipt*_ is a region-specific coefficient that relates the regional change in emissions of pollutant *p* to the change in average global forcing.

We used the MAGICC6 climate model^55^ to derive the coefficients by determining the impact on forcing from a pulse change in emissions in each time period in each region:

20 $$r_{ipt} = \left[ {\frac{{\partial {\mathrm{RF}}_t^{{\mathrm{aer}}}}}{{\partial {\mathrm{E}}_{itp}}}} \right]_{{\mathrm{MAGICC6}}}.$$

For this we ran MAGICC6 with a pre-defined representative concentration pathway (RCP) scenario and then again after having reduced the emissions of one pollutant in one region by a marginal amount. We repeated this procedure for each region, pollutant, time-step, and, finally, each RCP. We thus derived a reduced-form surface-response representation of aerosol forcing in MAGICC6. We separated the effects of the different pollutants to allow them to be controlled independently, as they are in reality (also see Supplementary Note 1). In this experiment, we observed a time dependence that accounts for changes in atmospheric dynamics as emissions accumulate:

21 $$r_{ipt} = u_{1,ip}\cdot t + u_{0,ip}.$$

The resulting coefficients incorporate both the direct and indirect forcing effects represented in MAGICC6, with the latter including those related to albedo and cloud responses. The time- dependence is independent of the initial conditions. We noted that *r* depends on the magnitude of the pulse change, but the effect is relatively small. Supplementary Table 3 reports values of *r*_*ipt*_ and Supplementary Fig. 7 shows the radiative forcing over time for three scenarios. Here the reader will note that we have used two separate models for estimating the health effects (TM5-FASST) and climate effects (MAGICC6) of air pollutant emissions, a decoupling that may introduce uncertainty. Nevertheless, MAGICC and TM5 have distinct strengths that we harness accordingly; MAGICC takes account of the full aerosol load of the whole atmosphere, top to bottom, and at the level of hemispheres, while TM5 tells us about the concentration at ground level, where people live and breathe, in principle at much higher resolution. Both models work with the same global emission inventories.

Aerosol forcing affects global mean atmospheric temperature just as CO_2_ forcing does, so that the added atmospheric temperature flow is equal to:

22 $$\Delta T_t^{{\mathrm{atm}}} = \xi ({\mathrm{RF}}_t^{{\mathrm{CO}}_2} + {\mathrm{RF}}_t^{{\mathrm{aer}}}),$$

where ${\mathrm{RF}}_t^{{\mathrm{CO}}_2}$ is CO_2_ forcing and *ξ* is the decadal speed of adjustment for atmospheric temperature (equal to 0.208).

### Aerosol feedbacks on the economy

As described, aerosol impacts occur from a change in radiative forcing and from a change in air quality. Changes in radiative forcing are transferred to RICE’s climate module where they influence the global average surface temperature, which is the basis of the monetized climate damage estimates^39^.

Changes in air quality are monetized as the health co-benefit, *B*, by multiplying the number of life years gained by the value of a life-year (VOLY). We follow the same approach taken in early versions of RICE’s climate damage function where a VOLY is assumed to equal 2 years of regional per capita consumption, *c*^39^:

23 $$B_{it}(\mu _{it},c_{it}) = {\mathrm{VOLY}}_{it}(c_{it}^{{\mathrm{pre - health}}})\cdot {\mathrm{LY}}_{it}^ \ast (\mu _{it},Y_{it},L_{it}).$$

A VOLY of 2 years of per capita consumption is generally the same order of magnitude as empirical estimates based on willingness-to-pay surveys^21^, but we also test several alternative values in sensitivity analyses. Final (post-health) per capita consumption, *c*_*it*_ is calculated as:

24 $$c_{it} = c_{it}^{{\mathrm{pre - health}}} + B_{it}/L_{it}.$$

With monetized aerosol impacts now included in the economic framework, RICE can follow its normal optimization procedure to find the decarbonization pathway that maximizes the objective (Eq. (1)).

### Additional information on select sensitivity analyses

The Supplementary Information contains additional information on the co-optimization of air quality and climate policies (Supplementary Note 1 and Supplementary Fig. 8), life-year monetization and valuation (Supplementary Note 2), integrating the FAIR climate module into RICE + AIR (Supplementary Note 3), and the development of FUND + AIR (Supplementary Note 4).

## Supplementary information

Supplementary Information

Description of Additional Supplementary Files

Supplementary Data 1

Supplementary Data 2

Supplementary Data 3

Supplementary Data 4

Supplementary Data 5

Supplementary Data 6

Supplementary Data 7

Supplementary Data 8

Supplementary Data 9

## Data Availability

The authors declare that all data supporting the findings of this study are available within the article and its Supplementary Information files.

## Code availability

The AIR model is licensed under the open source MIT license. The code is freely available at https://github.com/Environment-Research/AIR.
