# Supplementary material for: The impact of human health co-benefits on evaluations of global climate policy
Source: Nat Commun. 2019 May 7;10:2095. doi: 10.1038/s41467-019-09499-x (PMC6504956; doi:10.1038/s41467-019-09499-x)
Supplement: Supplementary file 1 — Supplementary Information [file 41467_2019_9499_MOESM1_ESM.pdf]

Supplementary Information for

The impact of human health co-benefits on evaluations of global climate policy

Scovronick et al.

Supplementary Figures

Note: "Reference case" refers to the model optimum where health benefits are excluded; aerosol co-reductions from CO_2 mitigation do occur, but only their climate impacts are included in the optimization. The reference case is therefore representative of standard cost-benefit climate-economy models. In contrast, "RICE+AIR" refers to the full model optimum that includes all climate and health impacts.

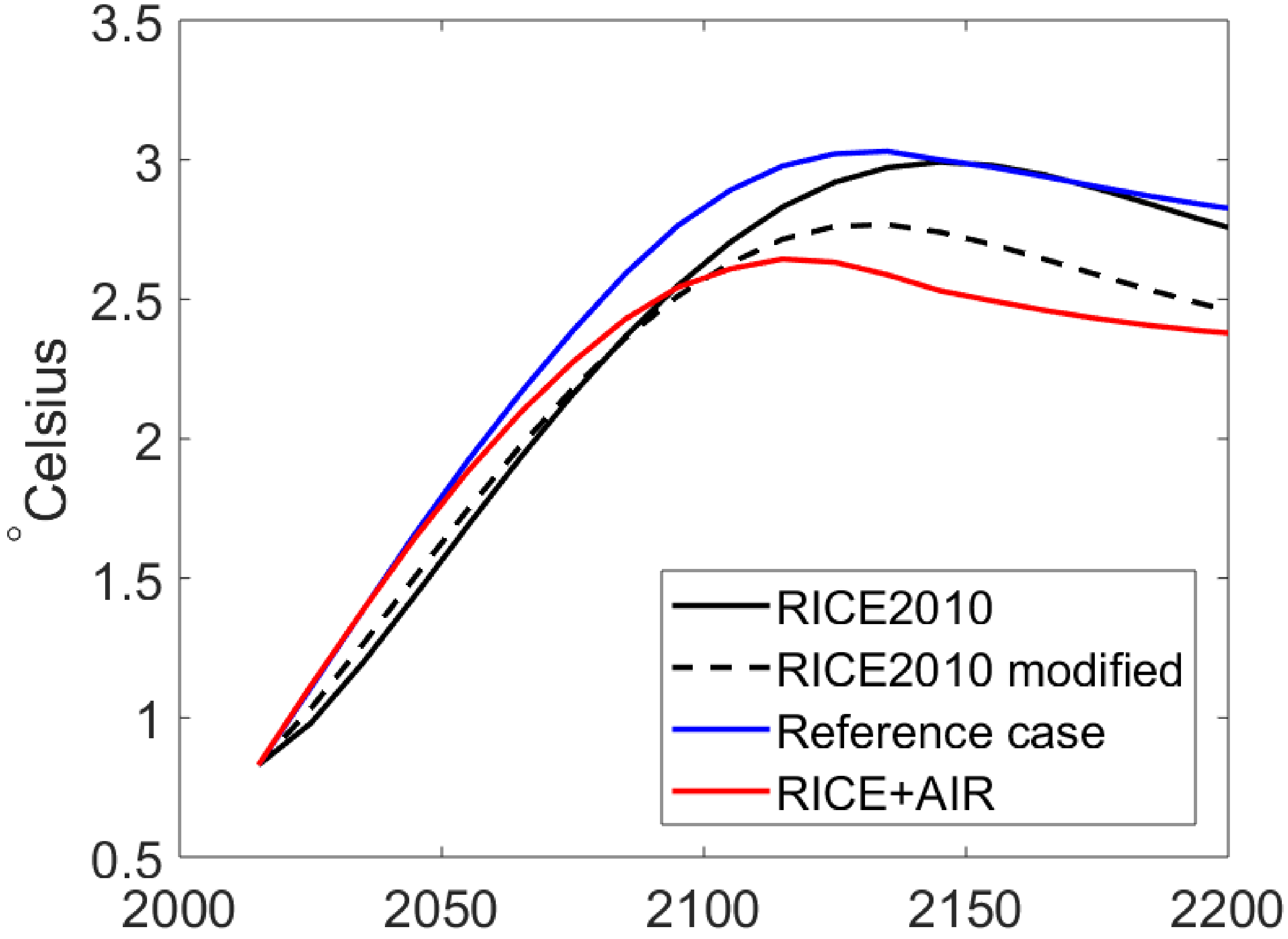

Supplementary Figure 1: Model comparison: RICE2010, Reference case, and RICE+AIR. The figure displays four optimal temperature trajectories that result from successive modifications to the unmodified, open-access Excel version of the RICE2010 model, culminating in the full RICE+AIR model that provides our main result:
1. RICE2010, which is the unmodified open-access Excel version of the model.
2. RICE2010 modified, which updates the population projections and modifies the social welfare function of RICE2010 (described in the Methods section) but maintains the exogenous aerosol assumptions.
3. Reference case, which adds endogenous aerosols but not health co-benefits to RICE2010 modified.
4. RICE+AIR, which adds health co-benefits to the Reference case and is the full model behind our main results.

.

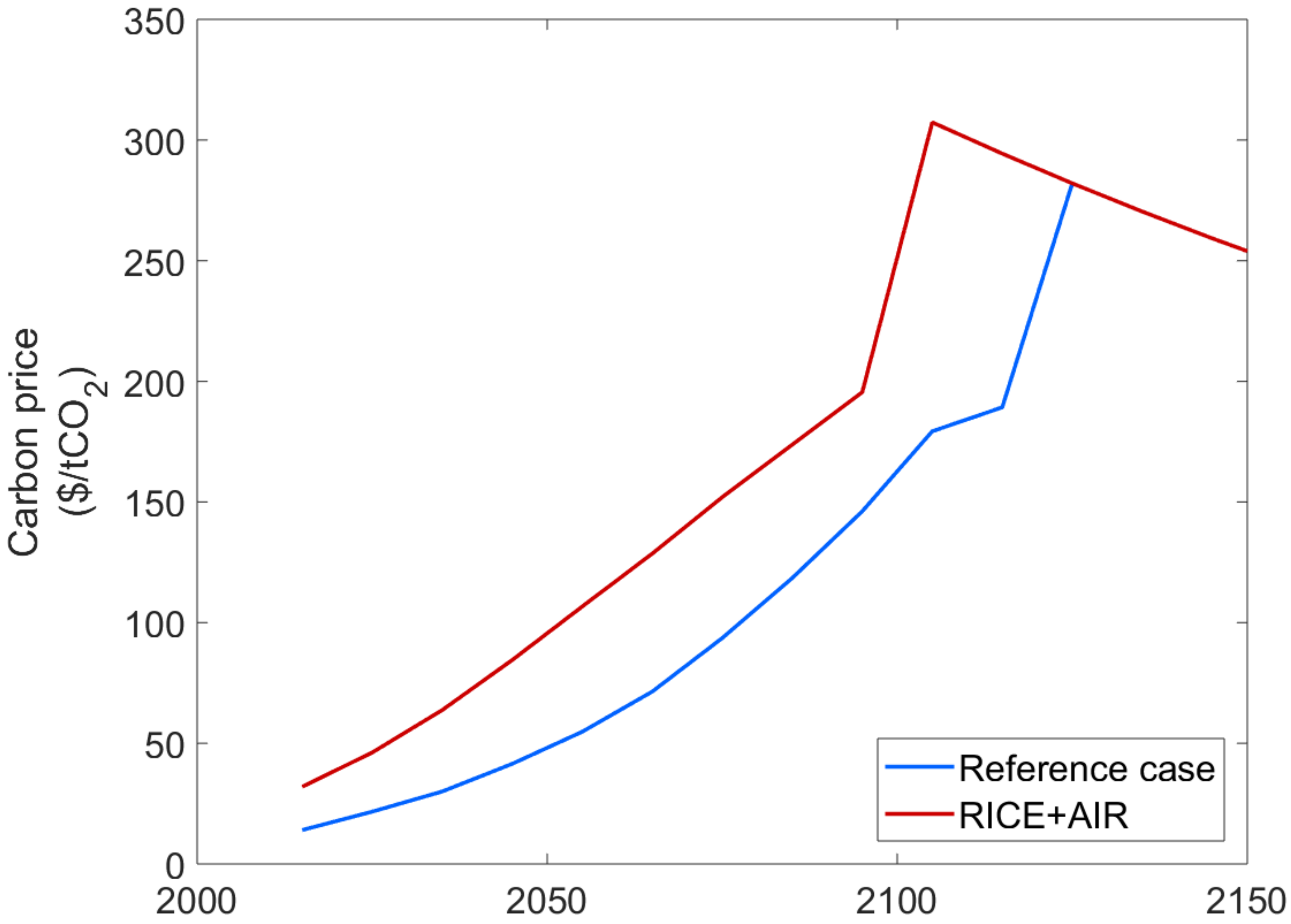

Supplementary Figure 2: Optimal carbon prices.
Carbon prices associated with the optimal decarbonzation and temperature trajectories reported in Figure 1 of the main text. (Note: full decarbonization occurs at the point where the carbon prices peak and subsequently decline along a single line, representing a backstop technology, which is a technology that can replace all fossil fuels).

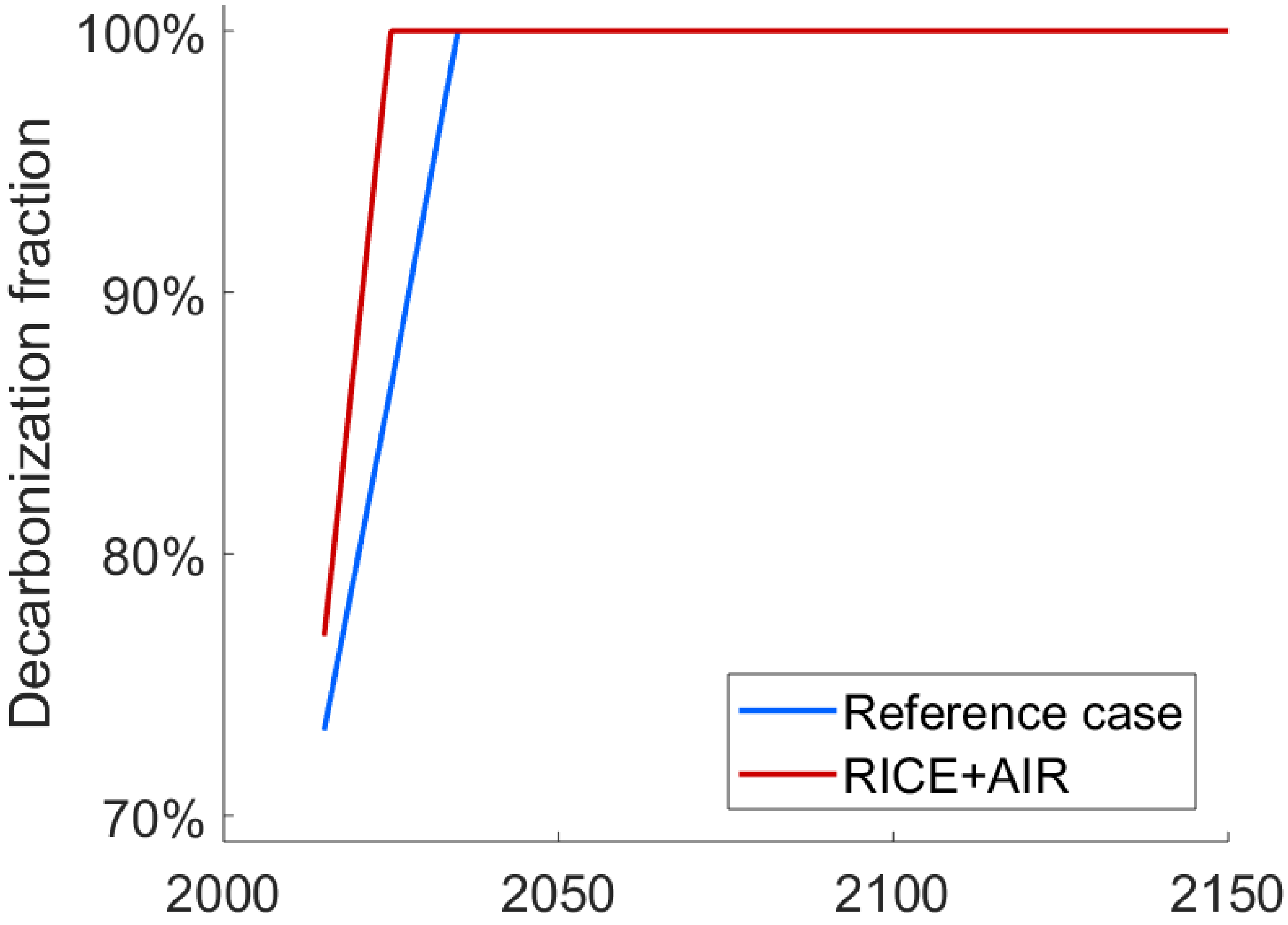

Supplementary Figure 3: Optimal decarbonization rates with a 3% discount rate. $\rho = 0, \eta = 1.3$.

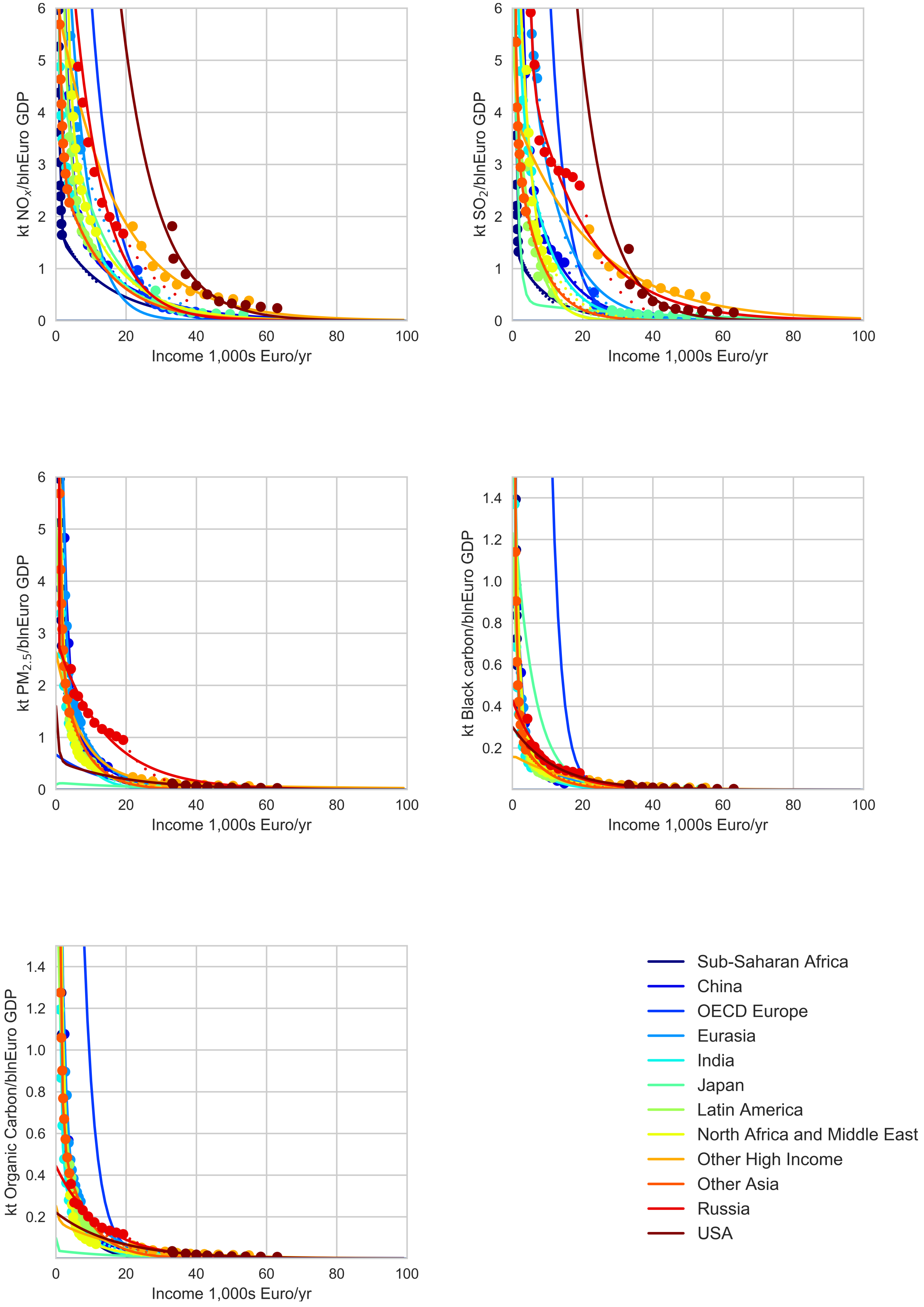

Supplementary Figure 4: Emission intensities by region and pollutant over time.

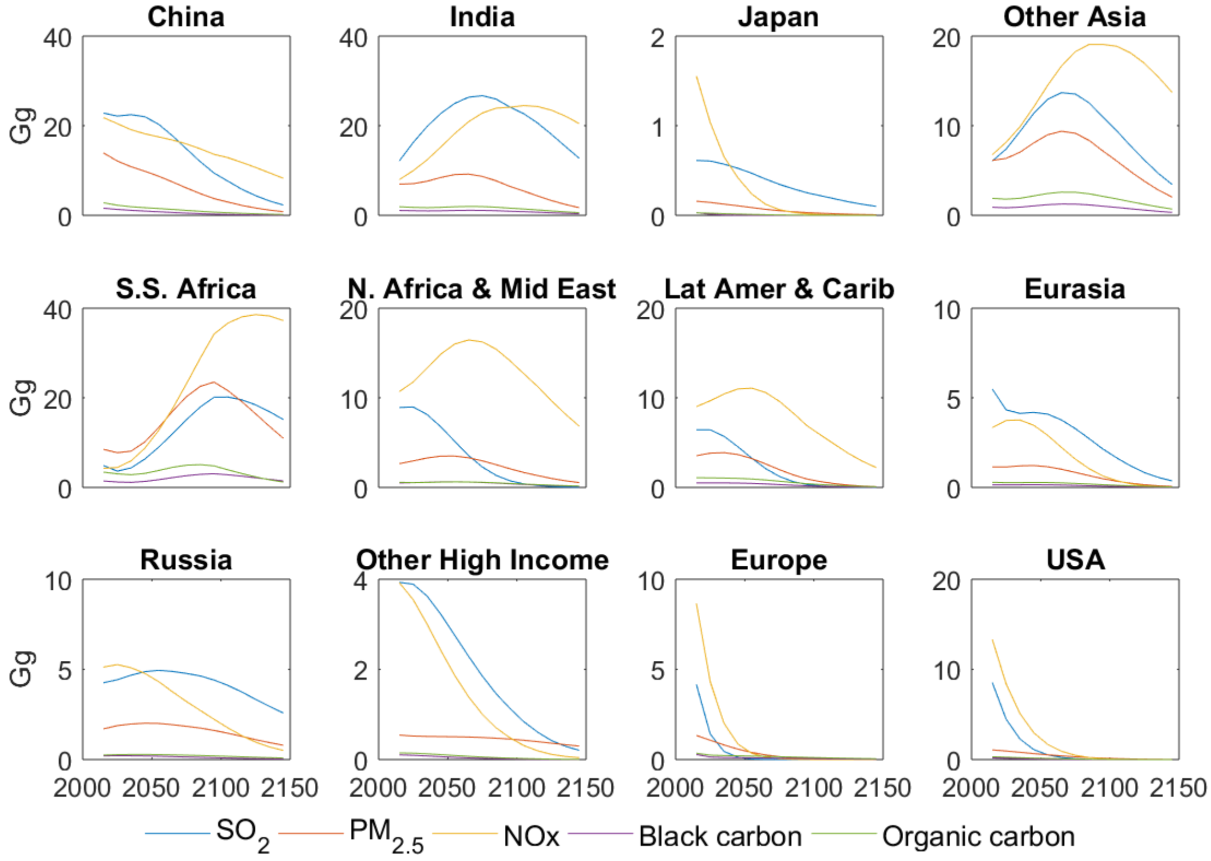

Supplementary Figure 5: Baseline (pre-mitigation) emissions by region.

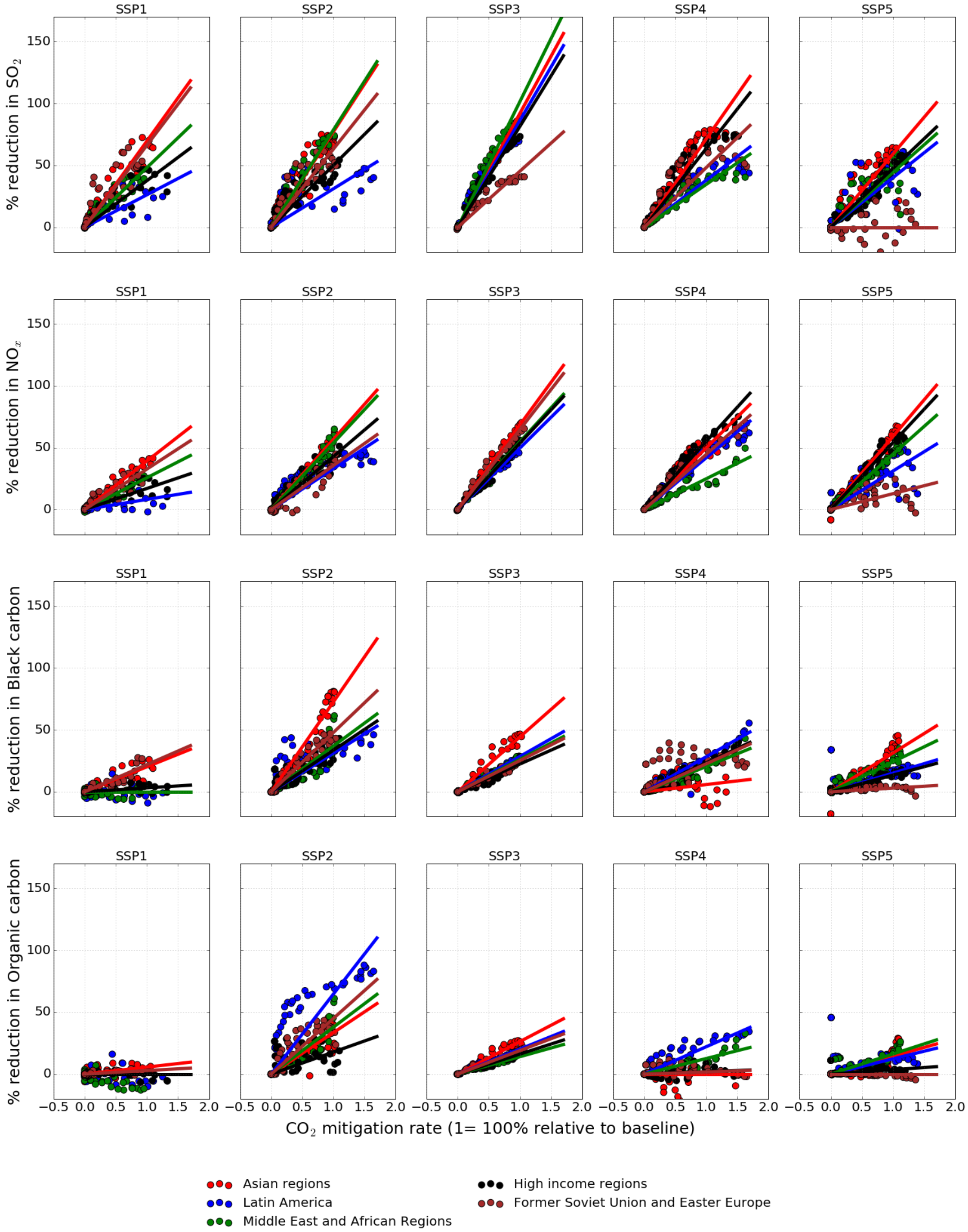

Supplementary Figure 6: Co-reduction relationships.
The slope of each line represents the fraction of air pollutant emissions that are reduced given a fraction reduction in CO_2 emissions. Each dot is a single region-specific estimate embedded within a given Shared Socioeconomic Pathway (SSP). The co-reduction relationship for $PM_{2.5}$ is taken as the average of black carbon and organic carbon.

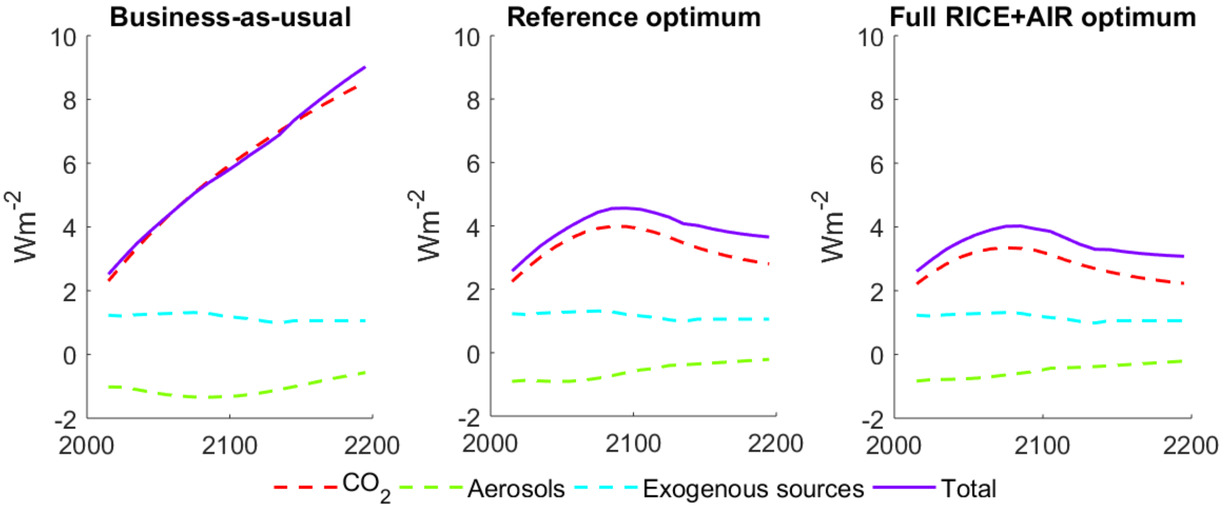

Supplementary Figure 7: Radiative forcing.
Radiative forcing over time in the business-as-usual scenario as well as the reference case and full RICE+AIR optima. Dotted lines sum to the solid purple line.

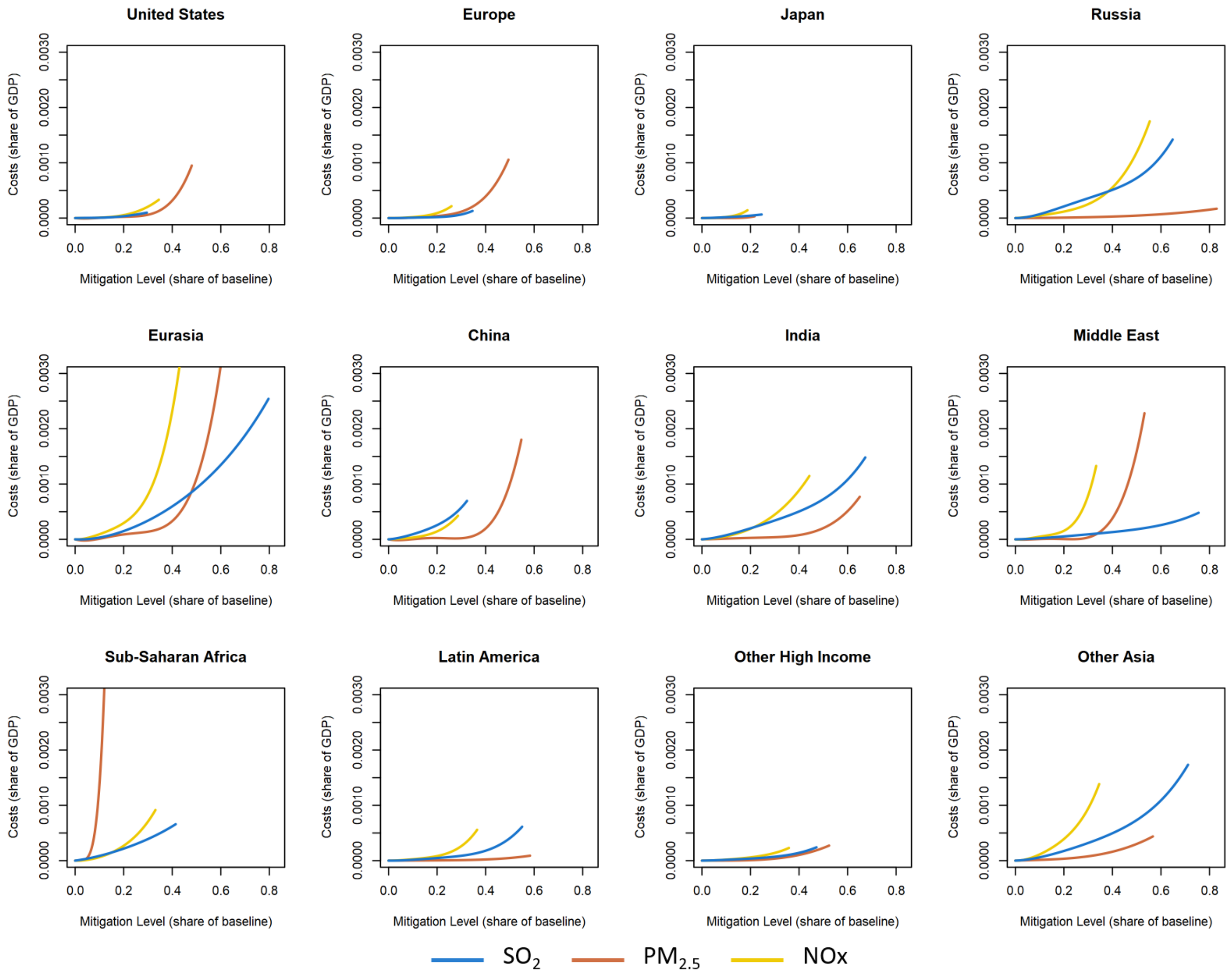

Supplementary Figure 8: Cost curves for air quality control.
Cost curves by region and pollutant used in the simulation where RICE+AIR optimizes on both climate policy and air quality policy simultaneously. The curves terminate at the maximum technologically feasible level of reduction that can be achieved through end-of-pipe measures.

Supplementary Tables

Supplementary Table 1: Effect of valuing life-years differently in wellbeing terms in the optimization. Percent increase in optimal decarbonization rates in 2030 and 2050 and the difference in peak temperature between the reference case and the RICE+AIR case using the following approaches (see Supplementary Note 2 for details):

- The approach underlying our main results where the value of a life year is higher in poor compared to rich regions.
- Three approaches where life-year values are equal across regions.
- An approach where the value of a life-year is higher in rich compared to poor regions. Specifically, the value of a life increases with the square root of consumption.

	2030	2050	Difference in peak temp (°C)†
Value richer less (main result)	52%	49%	0.39
Equal value 1*	30%	33%	0.38
Equal value 2‡	42%	45%	0.45
Equal value 3**	32%	37%	0.33
Value richer more	17%	26%	0.27

† Positive values indicate lower temperatures in the RICE+AIR case. Values are rounded.

* Inequality aversion (η) = 1, pure rate of time preference = 1.5.

‡ Inequality aversion (η) = 1, pure rate of time preference = 2.

** The value of a life-year is $\alpha * c^{\eta}$ (see Supplementary Note 2).

Supplementary Table 2: The modified objective function. Tables S5a and S5b show the percent change in results between the reference case and the full RICE+AIR case using two model versions that differ only in their objective function. "Model 1" is the model we implement whereas 'Model 2" is identical except it uses the standard RICE2010 objective function. The difference in the two objective functions is described below the table. Unlike Tables S5a and S5b, which are within-model comparisons, Table S5c compares results between the two models in the full RICE+AIR case of each. Together, the tables show that although our version of RICE recommends slightly faster decarbonization than the original version of the model, within both models the effect of accounting for health co-benefits and aerosol forcing is similar in magnitude.

Table S5a: Within-model comparison: Optimal decarbonization				
	2030	2050	2070	2090
Model 1	52%	49%	39%	22%
Model 2	48%	44%	35%	20%
Table S5b: Within-model comparison: Temperature rise				
	2030	2050	2070	2090
Model 1	<1%	-2%	-5%	-8%
Model 2	<1%	-2%	-3%	-6%
Table S5c: Between-model comparison: Percent change from Model 1 to Model 2 in the RICE+AIR case				
	2030	2050	2070	2090
Decarb rate	-17%	-17%	-17%	-15%
Temperature	<-1%	1%	2%	5%

The modified objective function explained

Consider the following general weighted objective function that is often used in climate-economy models:

$$W(c_{it}) = \sum_{it} \aleph_{it} \frac{L_{it}}{(1+\rho)^{10t}} \frac{c_{it}^{(1-\eta)}}{1-\eta} \tag{S1}$$

If the weights that appear in this equation, $\aleph$, are proportional to the inverse of the marginal utility of consumption they are called Negishi weights. Negishi weights are used in many climate-economy models, including early versions of RICE, where they were introduced to impose constraints on capital flows.[1] RICE2010 does not require weights for this purpose (because regions are autarkic), but they are still used so that the maximization as market simulation principle holds [2].

Our objective function (Equation 1 in the Methods) is identical to Equation S1 but without any weights ($\aleph = 1$). This is because we do not suppose our results represent a market simulation since we do not expect the mitigation rates to emerge as the result of an unregulated market. Furthermore, Negishi weights distort time preferences [3] and the inter-regional trade-off [4] in ways that are opaque and difficult

[1]The first version of RICE was implemented like a computational general equilibrium model, in which there would be capital flows until the marginal utilities of consumption are equated across regions. See [1].

to justify, both descriptively and normatively. We have explained this change in more detail previously [5].

Supplementary Table 3: Radiative forcing coefficients by region in 2050 and 2100 (W/m^{-2}) per Gg emissions).

	SO_2	NOx	Black carbon	Organic carbon
		**2050**		
USA	-0.0121	-0.0052	0.0739	-0.0088
Europe	-0.0121	-0.0052	0.0739	-0.0088
Japan	-0.0121	-0.0052	0.0739	-0.0088
Russia	-0.0129	-0.0048	0.0732	-0.0082
Eurasia	-0.0129	-0.0048	0.0732	-0.0082
China	-0.0112	-0.0056	0.0746	-0.0095
India	-0.0112	-0.0056	0.0746	-0.0095
North Africa and Middle East	-0.0094	-0.0065	0.0760	-0.0109
Sub-Saharan Africa	-0.0094	-0.0065	0.0760	-0.0109
Latin America	-0.0067	-0.0077	0.0780	-0.0129
Other High Income	-0.0121	-0.0052	0.0739	-0.0088
Other Asia	-0.0112	-0.0056	0.0746	-0.0095
		**2100**		
USA	-0.0130	-0.0062	0.0726	-0.0103
Europe	-0.0130	-0.0062	0.0726	-0.0103
Japan	-0.0130	-0.0062	0.0726	-0.0103
Russia	-0.0140	-0.0058	0.0718	-0.0097
Eurasia	-0.0140	-0.0058	0.0718	-0.0097
China	-0.0121	-0.0065	0.0733	-0.0109
India	-0.0121	-0.0065	0.0733	-0.0109
North Africa and Middle East	-0.0101	-0.0072	0.0748	-0.0122
Sub-Saharan Africa	-0.0101	-0.0072	0.0748	-0.0122
Latin America and Caribbean	-0.0071	-0.0082	0.0771	-0.0140
Other High Income	-0.0130	-0.0062	0.0726	-0.0103
Other Asia	-0.0121	-0.0065	0.0733	-0.0109

Supplementary Note 1: Co-optimizing air quality and climate policy

Our main results assume that autonomous air quality control proceeds approximately as expected. However, in addition to exogenously specifying such control, there is capability in AIR to cost-optimize it through end-of-pipe measures. In this case, RICE+AIR selects the optimal combination of both air quality and climate policies. Upper limit emission reductions and associated cost curves for each pollutant and region are derived and projected based on data from the GAINS model using the maximum technically feasible reduction scenarios (Supplementary Figure 8). We deflate the technology costs in GAINS by 50% to estimate the total costs to the economy, which are generally lower than technology costs [6]. Note that climate policy can reduce air pollutant emissions below what is technically feasible through end-of-pipe technologies alone — for example by switching from a coal plant to solar photovoltaic — and therefore may still lead to co-benefits even in regions that implement all available end-of-pipe measures. We assume that end-of-pipe air pollution technologies do not affect CO_2 emissions.

Supplementary Note 2: Life-year monetization and valuation

Our main results assume that an additional life-year lived is proportional to regional per capita consumption. One possible concern with this approach, where the monetization method is based on regional per capita consumption, is that it may appear to imply that an additional life-year lived counts for less in a poorer region than in a richer region. However, our modeling avoids this worrisome implication because of the degree of concavity in the objective function in consumption (Equation 1 in the Methods). This concavity, represented by the elasticity of marginal utility, η, results in diminishing marginal utility of consumption (inequality aversion) in the objective function. Specifically, impacts are valued differently depending on the consumption level, so that an impact of x dollars is counted in the objective as $x * {c_i}^{-\eta}$ when it affects an individual with consumption c_i. As a result, the wellbeing value of a life year (WLY) to a person with consumption c_i is computed as follows:

$$WLY = VOLY * c_i^{-\eta} = 2c_i^{(1-\eta)} \tag{S2}$$

To reiterate, the $VOLY$ is what a life-year is worth in dollar terms whereas the WLY is the wellbeing value of the VOLY after it passes through the objective function. Equation S2 shows that when $\eta > 1$, each life-year is valued more highly in the objective function in poor regions than in rich regions. As a result, because the standard value of η we use is 1.5, our objective function actually assigns greater value to each life-year gained in poorer regions, despite each life-year having a lower monetized value in those regions.

With this in mind, we designed a further sensitivity test (Supplementary Table 1) to explore the

implications of alternative approaches that each ensure that life-years are weighted exactly equally in rich and poor regions in the objective function, despite having different monetized VOLYs. The first is to set η = 1, which has the desired effect; however, since this method affects the objective function generally, it has the broader influence of increasing optimal decarbonization independent of the health impacts. Therefore, in the second approach we compensate for this issue by also adjusting the rate of time preference so the reference case with $\eta = 1$ approximates the standard reference case with $\eta = 1.5$. For the final approach, we run the model with the assumption that the value of a life-year is $\alpha * c^{\eta}$. By Equation S2 this leads to a social value of a life-year that is equal to α. In particular, it is identical across income groups. We choose α so that the total discounted social value of the health co-benefits are equal to what they are when $VOLY = 2c$ under the full RICE+AIR model optimum.

In contrast and in addition to these sensitivites, Supplementary Table 1 also reports results when rich lives are valued more than poor lives in the objective function by setting $VOLY{=}\alpha * c^2$. By Equation S2 this results in a social value of a life-year that is equal to $\alpha * \sqrt{c}$, which is increasing in income (i.e. the social value of a life increases with the square root of consumption). Here again we choose α so that the total discounted social value of the health co-benefits are equal to what they are when $VOLY = 2c$ under the full RICE-AIR model optimum.

Results of all sensitivity analyses described in this section show that the strong effect of adding health co-benefits remains under all these approaches, although it is generally somewhat reduced.

Supplementary Note 3: The FAIR climate model

In one sensitivity presented in the main text we performed an experiment where we ran RICE+AIR using the Finite Amplitude Impulse Response (FAIR) climate model [7], version 1.0, as an alternative to RICEs native climate model. However these two models are not straightforwardly compatible, in large part because RICE runs on decadal time steps while FAIR is annual. Creating yearly versions of the socioeconomic, emission, and climate damage modules from RICE would address this issue, but such an exercise requires significant model development and validation efforts beyond the scope of this paper.

We instead created an iterative version of RICE+AIR with FAIR that simulates a direct coupling between the two models. This iterative approach proceeds in several steps:

1. For a given global carbon tax, use a deterministic version of RICE+AIR to calculate CO_2 emissions and aerosol radiative forcing values.

2. Interpolate the CO_2 and aerosol radiative forcing decadal values into annual values.

3. Use these interpolated results and the same exogenous radiative forcing scenario to calculate projected temperatures with FAIR (we import the aerosol forcing estimates from RICE+AIR because endogenous aerosol forcing is not represented FAIR version 1.0).

4. Run RICE+AIR with each decadal value from the updated FAIR temperature projections, which changes the estimated climate damages, economic output, and CO_2 emission levels.

5. Use these updated CO_2 emission and aerosol forcing values from RICE+AIR to again run FAIR.

We repeat the process outlined above until the results converge and can thus optimize this iterative version of the model.

Supplementary Note 4: FUND+AIR

Like RICE/DICE, FUND is one of the three leading climate-economy models used by the US Interagency Working Group to estimate the social cost of carbon [8]. (The third model, PAGE, is less widely used). FUND is a 16-region global model that, unlike RICE, was not designed to optimize. Instead, FUND is mainly used to either explore the impacts of climate policy scenarios or compute the stream of climate-related damages from a pulse emission of CO_2. FUND has different world regions, a different economic framework, a different climate module and a different specification of climate damages when compared to RICE. It thus provides an important opportunity to explore model uncertainty. The key features of FUND have been described elsewhere [9, 10]; here we limit the discussion to the modifications we made to optimize the model and link it to the AIR module.

Optimization: We optimized FUND through 2150 by finding the carbon tax pathway that maximizes Equation 1 of the Methods, with the same basic trade-off as in RICE between mitigation costs and climate damages. Results were computed with the same discounting parameters used in the RICE+AIR model runs – an inequality aversion and time preference of 1.5 and 1.5%, respectively.

Adding the AIR module: We implemented FUND+AIR through the same approach described above for RICE+AIR, as summarized in two steps. First, we removed all exogenous aerosols and their precursors, and endogenized the (pre-mitigation) emissions (see Equations 2-4 in the Methods section). Second, we allowed emissions to change as a result of CO_2 mitigation (Equation 6 in the Methods section). The difference in pre- and post-mitigation emissions determines the impact of decarbonization on radiative forcing and human health using the same methods described above for RICE+AIR (Equations 8-23). All relevant parameters were fit to the FUND regions, which differ somewhat from the RICE regions. For consistency with RICE+AIR, each life-year gained was valued at two years of per capita consumption.

Supplementary References

[1] W. D. Nordhaus and Z. Yang, "A regional dynamic general-equilibrium model of alternative climate-change strategies," *The American Economic Review*, pp. 741–765, 1996.

[2] W. Nordhaus and P. Sztorc, "Dice 2013R: Introduction and user's manual," 2013.

[3] F. Dennig and J. Emmerling, "A note on optima with Negishi weights." 2017.

[4] E. A. Stanton, "Negishi welfare weights in integrated assessment models: the mathematics of global inequality," *Climatic Change*, vol. 107, no. 3-4, pp. 417–432, 2011.

[5] F. Dennig, M. B. Budolfson, M. Fleurbaey, A. Siebert, and R. H. Socolow, "Inequality, climate impacts on the future poor, and carbon prices," *Proceedings of the National Academy of Sciences*, vol. 112, no. 52, pp. 15827–15832, 2015.

[6] Z. Vrontisi, J. Abrell, F. Neuwahl, B. Saveyn, and F. Wagner, "Economic impacts of eu clean air policies assessed in a cge framework," *Environmental Science & Policy*, vol. 55, pp. 54–64, 2016.

[7] R. J. Millar, Z. R. Nicholls, P. Friedlingstein, and M. R. Allen, "A modified impulse-response representation of the global near-surface air temperature and atmospheric concentration response to carbon dioxide emissions," *Atmospheric Chemistry and Physics*, vol. 17, no. 11, pp. 7213–7228, 2017.

[8] Interagency Working Group on Social Cost of Greenhouse Gases, *Technical Update of the Social Cost of Carbon for Regulatory Impact Analysis Under Executive Order 12866*. United States Government, 2016.

[9] R. S. Tol, "On the optimal control of carbon dioxide emissions: an application of fund," *Environmental Modeling and Assessment*, vol. 2, no. 3, pp. 151–163, 1997.

[10] S. T. Waldhoff, D. Anthoff, S. Rose, and R. S. Tol, "The marginal damage costs of different greenhouse gases: An application of fund," *Economics*, vol. 8, pp. 1–33, 2014.
