# Supplementary material for: The impact of human health co-benefits on evaluations of global climate policy
Source: Nat Commun. 2019 May 7;10:2095. doi: 10.1038/s41467-019-09499-x (PMC6504956; doi:10.1038/s41467-019-09499-x)
Supplement: Supplementary file 2 — Description of Additional Supplementary Files [file 41467_2019_9499_MOESM2_ESM.pdf]

Description of Additional Supplementary Information

File Name: Supplementary Data 1
Description: Data underlying Figure 1

File Name: Supplementary Data 2
Description: Data underlying Figure 2

File Name: Supplementary Data 3
Description: Data underlying Figure 3

File Name: Supplementary Data 4
Description: Data underlying Figure 4

File Name: Supplementary Data 5
Description: Data underlying Figure 5

File Name: Supplementary Data 6
Description: Data underlying Figure 6

File Name: Supplementary Data 7
Description: Data underlying Table 1

File Name: Supplementary Data 8
Description: Data underlying Table 2

File Name: Supplementary Data 9
Description: Data underlying Table 3
